# Ultrathin, Unsinkable, Janus‐Faced Solar–Thermal Interfacial Evaporator for High‐Throughput Seawater Distillation and Solar‐Water Production

**DOI:** 10.1002/advs.202511600

**Published:** 2025-10-21

**Authors:** Mohammed Aslam Villan, Amrutha Suresh, Mukund Misra, Cintia Naranjo Ruiz, Neil R. Cameron, Sandip K. Saha, Subramaniam Chandramouli

**Affiliations:** ^1^ Department of Chemistry Indian Institute of Technology Bombay Mumbai Maharashtra 400076 India; ^2^ Department of Materials Science and Engineering Monash University 14 Alliance Lane Clayton VIC 3800 Australia; ^3^ Department of Mechanical Engineering Indian Institute of Technology Bombay Mumbai Maharashtra 400076 India; ^4^ Instituto de Química Pontificia Universidad Católica de Valparaíso Avenida Universidad 330 Curauma Valparaíso 00000 Chile; ^5^ School of Engineering University of Warwick Coventry CV4 7AL UK; ^6^ Nanotechnology and Catalysis Research Centre (NANOCAT) Universiti Malaya Kuala Lumpur 50603 Malaysia

**Keywords:** nanocarbon materials, salt rejection, solar–thermal interfacial evaporation, water desalination, water‐energy nexus

## Abstract

Solar–thermal distillation offers immense and hitherto untapped potential for energy‐efficient, sustainable freshwater production. However, critical limitations in material and system design continue to hinder water‐evaporation rate (*R*_w_) and specific water productivity (SWP), posing significant roadblocks for scalable and stable solar desalination. An ultrathin, porosity‐tuned high internal phase emulsion polymer (polyHIPE, PH) scaffold is integrated with nanostructured hard‐carbon florets (NCF) as a black‐body absorber to create the thinnest interfacial evaporator, delivering best‐in‐class solar–thermal energy conversion (*η*‐STC = 84%), rapid *R*_w_ (6.5 kg m^−2^ h^−1^), and efficient SWP of 18 L m^−2^ day^−1^ from seawater. The Janus‐faced NCF@PH confines solar–thermal energy at the interface, which also promotes extensively convex water menisci, to deliver high *R*_w_. The continuously interconnected 3D capillary channels within NCF@PH ensure continuous water transport, while their hydrophobic surfaces prevent salt deposition for robust long‐term seawater distillation with high salt rejection (>90%). Furthermore, a system‐level prototype termed SunSpring (SS) works to decouple the light‐admitting, water‐evaporation, and water‐condensation steps to produce environmental protection agency (EPA)‐standard freshwater (<10 ppm salinity) from highly saline seawater (35 000 ppm) for over ≈225 h of real‐time continuous usage. It is also estimated that SS has the lowest energy (<3 W L^−1^) and CO_2_ (≈3 g L^−1^) footprints, representing a paradigm shift in the water‐energy nexus.

## Graphical abstract

A nanostructured carbon‐coated porous polymeric scaffold is developed to accelerate the hydrological cycle, enabling solar–thermal freshwater production from saline water with reduced energy consumption and CO_2_ footprint.

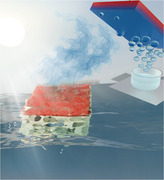

## 1 Introduction

The growing scarcity of clean water remains a persistent global challenge that is further intensified by the nonuniform distribution of electrical energy necessary for its purification.^[^ ^1^, ^2^, ^3^, ^4^, ^5^ ^]^ Water‐energy nexus has emerged as a critical factor for transitioning to sustainable development, with over 400–1200 TWh year^−1^ of energy consumed globally for producing potable water from oceans and underground water sources.^[^ ^6^, ^7^, ^8^, ^9^ ^]^ In this context, desalination of water to remove dissolved salts using techniques such as reverse osmosis (RO), ultra‐ and nanofiltration (NF), and electrodialysis (ED) offers possible solutions to tackle the increasing water scarcity.^[^ ^2^, ^10^, ^11^, ^12^, ^13^ ^]^ However, all these techniques are energy intensive and utilize ≈3–7 W h for every liter of purified water.^[^ ^14^, ^15^, ^16^, ^17^ ^]^ Moreover, the large volume and high salinity of water reject from such technologies (≈30–60% containing 2–3 times the initial total dissolved solids, TDS) raises serious concerns on environmental sustainability.^[^ ^18^, ^19^ ^]^

Fundamentally, all these techniques are based on removing the dissolved salts from water, accounting for the high‐energy footprint and water rejection. On the other hand, techniques such as multistage flash distillation and low‐temperature thermal desalination operate to remove water from the dissolved salts.^[^ ^20^, ^21^ ^]^ Again, the energy‐intensive nature of these processes makes it unviable, leading to their poor worldwide adaptability (<1%).^[^ ^22^, ^23^, ^24^ ^]^

Nearly 30% of the global population lives at the intersection of high water stress, economic backwardness, and abundant solar irradiance (>5 kWh m^−2^ day^−1^). Leveraging clean sunlight to evaporate water followed by its condensation, solar–thermal water distillation utilizes the least electrical power for operation and offers single‐step removal of suspended particulates, dissolved salts, and pathogens.^[^ ^25^, ^26^ ^]^ Thus, solar stills offer immense potential and promise for achieving comprehensively and equitable access to clean water globally, with the capability to handle varied water sources while maintaining a low carbon footprint. However, a major drawback has been its sluggish kinetics of water evaporation and related inefficiencies.^[^ ^12^, ^27^, ^28^, ^29^, ^30^ ^]^ These challenges have recently received a major fillip from attempts to synergize advanced materials for interfacial solar–thermal conversion (STC) with system design that increases the overall thermal efficiency of the process.^[^ ^28^, ^31^, ^32^, ^33^, ^34^, ^35^, ^36^, ^37^, ^38^, ^39^, ^40^ ^]^ Consequently, the enhanced kinetics of mass transfer and localized improved heat transfer have resulted in enhancing the rate of water evaporation (*R*_w_ ≈ 3–6 kg m^−2^ h^−1^)^[^ ^35^, ^41^, ^42^, ^43^, ^44^ ^]^ and thermal efficiency (40–50%), compared to conventional solar stills (0.5–2.0 kg m^−2^ h^−1^, 10–15%).^[^ ^45^, ^46^, ^47^ ^]^ In this context, materials ranging from nanocarbon‐coated cellulose‐based fabrics,^[^ ^34^, ^48^ ^]^ nanocarbon‐hydrogel hybrids,^[^ ^39^, ^49^ ^]^ bioderived carbons,^[^ ^50^, ^51^, ^52^ ^]^ to nanoscale plasmonic absorbers^[^ ^53^, ^54^ ^]^ have been utilized for enhancing the water‐evaporating rate. Synergistic coupling of such photothermal materials with innovative membranes that resist salt deposition has gained increasing attention.^[^ ^55^, ^56^, ^57^, ^58^ ^]^ These innovations have been coupled to system‐level engineering that apparently lowers the latent heat of vaporization (∆*H*_LV_) and simultaneously increases the water meniscus to realize high *R*_w_, the exact mechanisms of which are still being investigated.^[^ ^59^, ^60^, ^61^, ^62^, ^63^, ^64^ ^]^

A majority of these studies have focused on the energy‐demanding water‐evaporation step. Consequently, the other important half of condensing the water vapor to recover pure water distillate has also been a major contributor for lowering the throughput of purified water. The exothermicity of water condensation results in heating the collector, leading to a self‐limiting process that slows the overall rate of water distillate.^[^ ^32^, ^34^, ^35^, ^48^, ^49^ ^]^ These limitations are also evident in real‐time studies that deliver a solar‐freshwater production (R_c_) of 0.15–0.4 Lm^2^ h^−1^, although the *R*_w_ values reported in these studies are substantially higher at 1.5–2 kg m^−2^ h^−1^. Along these lines, a double‐layered floating vertical enclosed evaporation system has been found to enhance the water‐condensation rate (*R*_c_) of 0.67 kg m^−2^ h^−1^ from water‐evaporation rate (*R*_w_) of 1.29 kg m^−2^ h^−1^ yielding a gained‐output ratio (GOR) of 0.51.^[^ ^38^ ^]^ A detailed analysis of several major breakthroughs reveal that the high *R*_w_ has been driven through material‐led innovations.^[^ ^36^, ^50^, ^51^, ^52^, ^53^, ^54^, ^59^, ^60^, ^65^, ^66^ ^]^ However, the *R*_c_ has been consistently poor or not reported, highlighting the challenges in synchronizing the two halves of the water‐purification cycle to enhance SWP.^[^ ^32^, ^61^, ^67^, ^68^ ^]^ Here, we adopt a comprehensive approach to look at design principles that enable a tailored approach for new materials delivering a) high solar–thermal conversion, b) enhanced water evaporation that is balanced with water transport and decoupled from, c) water collection with, d) good salt rejection (SR) to achieve e) stability of operation in highly saline environments. Importantly, this work seeks to complete the water cycle by establishing routes that capture the evaporated water vapor to produce fresh water with increased thermal and energy efficiencies.

An ideal material for efficient STC requires strong broadband absorption (*α* → 1), good crystallinity for high phonon density of states and strong phonon activation, while exhibiting low thermal conductivity and emissivity (*ε* → 0) to minimize parasitic heat losses.^[^ ^69^, ^70^ ^]^ Such a material should also exhibit high thermal effusivity for enhanced and directed heat transfer to the interfacial water. Water transport within such an interfacial scaffold demands porous and hydrophilic channels, while hydrophobic surfaces are preferred to minimize salt crystallization, fouling, and mechanical disintegration of the membrane.^[^ ^25^, ^26^, ^31^, ^65^ ^]^ Moreover, a multilayered material for effective heat localization would pose challenges like interfacial delamination and sinking on long‐term usage. Thus, an ideal interfacial water evaporator has to be Janus‐faced with an appropriate engineered combination of hydrophobic and hydrophilic porous and chemically stable channels to ensure seamless water transport and simultaneously have low density to permanently float at air–water interface.^[^ ^31^, ^61^ ^]^ Furthermore, the material design should also enable thermal management by localizing the heat within the interfacial layer.^[^ ^39^, ^71^, ^72^, ^73^ ^]^ Incorporating all these rigorous and contrasting demands and simultaneously integrating the material with a suitable support has been challenging and highly desirable. Finally, the effective collection of the water vapor demands a hydrophobic surface that is kept consistently cooler than the surroundings to enhance the formation and rapid roll‐down of water droplets. Materials, approaches, and design principles that address all these challenges to achieve energy‐efficient, reliable, and affordable solar–thermal water desalination with high throughput of pure water are both important and an immediate requirement.^[^ ^40^, ^74^, ^75^, ^76^ ^]^

Addressing these fundamental and practical challenges, we present strategic design principles for seamless integration of such mutually exclusive and strongly anticorrelated properties through careful combination of complementary materials. An excellent solar–thermal convertor—nanocarbon florets (NCF)—is integrated over porosity‐engineered hydrophobic high internal phase emulsion polymer (polyHIPE, PH) sheets to create an unsinkable, ultrathin (200 µm) solar–thermal evaporator (NCF@PH) that achieves an excellent *η*‐STC of 84% and a surface temperature (*T*_s_) of 120 °C, with an *R*_w_ of 4.5–6.5 kg m^−2^ h^−1^ under widely varying saline environments (35k–0k ppm TDS), including simulated seawater. Importantly, the NCF@PH is the thinnest such interfacial evaporator and overcomes fundamental tradeoff to achieve excellent *R*_w_ and *η*‐STC, outperforming several other materials in the domain. We present detailed experimental evidence of NCF@PH achieving a) heat localization within the interfacial water layer, with negligible parasitic loss, b) enhanced proportion of intermediate water (IW) within its continuously interconnected pore structure that also c) drives the formation of multiple water menisci to d) ensure fine balance between water transport and *R*_w_, and e) avoid pore blocking through salt deposition during its operation in highly saline seawater like environments (10–35k ppm TDS). 3D X‐ray tomographic and elemental mapping images establish negligible salt deposition within the hydrophobic water‐transport channels of NCF@PH, yielding high salt rejection (≈90% at 35k ppm TDS). Moreover, complete reusability and regeneration of the NCF@PH are demonstrated by facile and rapid removal of the localized salt deposition over its exposed surface. In continuation, we decouple the light‐admitting surface and the water‐condensing surface by incorporating a Peltier‐cooled hydrophobic water condenser that drives rapid water droplet formation and roll‐out to achieve exceptionally a high SWP of ≈18 L m^−2^ day^−1^ with robust operation over 225 solar hours in seawater conditions (35k ppm).

## 2 Results and Discussion

### 2.1 Design of Ultrathin Scaffold of the Interfacial Evaporator

**Figure 1:**
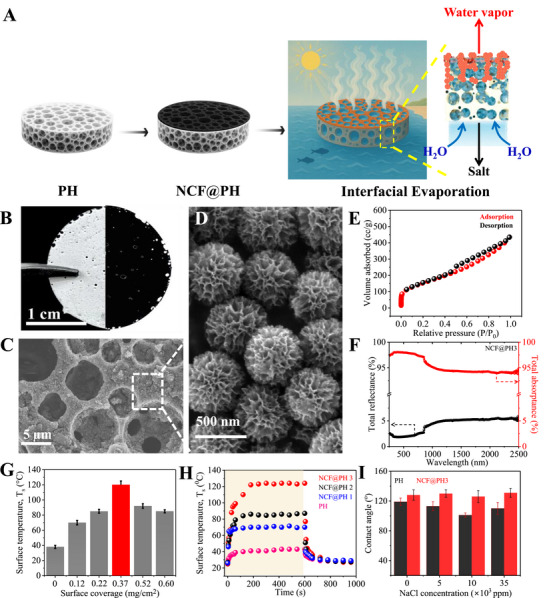
Design and integration of solar–thermal interfacial evaporator. A) Schematic representation of various steps for the fabrication of NCF@PH solar–thermal interfacial evaporator, starting from pristine PH over which NCF is spray‐coated for the final assembly of the setup. B) Photograph of pristine PH membrane (left, white) and NCF@PH (right, black). C) Scanning electron microscopy (SEM) image of NCF@PH, with a magnified region, D) establishing the presence of NCF. E) N_2_ gas adsorption isotherm of NCF recorded at 77 K. F) Total reflectance and absorptance spectra of NCF@PH over the entire solar spectrum from 250 to 2500 nm. G) Variation in surface temperature (*T*_s_) of NCF@PH under different loadings (mg cm^−2^) of NCF, as estimated through thermometric imaging. H) Temporal evolution of *T*_s_ for different surface coverage of NCF over PH, in the range of 0.00–0.37 mg cm^−2^. I) Average contact angle of deionized water and salt water at different salinities (5000–35 000 ppm, NaCl) on the surface of pristine PH and NCF@PH3.

Achieving a Janus‐faced interfacial evaporator with the graded surface wettability is the first key requirement for achieving high evaporation rate.^[^ ^31^ ^]^ To this end, high internal phase emulsion polymerization‐enabled precise homopolymerization of trimethylolpropane triacrylate (TMPTA) along with controlled heteropolymerization of trimethylolpropane tris(3‐mercaptopropionate) (TMPTMP) to yield shapeable porosity‐engineered PH monolithic sheets (see Figure S1 in the Supporting Information).^[^ ^77^, ^78^, ^79^, ^80^ ^]^ The PH exhibits well‐defined, interconnected pore structure, and uniform distribution of surface thiol groups (**Figure** **1**A,B; Figure S2a–f, Supporting Information). Such a PH scaffold incorporates several desirable properties such as a) hierarchically interconnected porous channels (80% porosity; see Figure S2e,f in the Supporting Information), b) strong covalent framework for salt resistance, mechanical robustness, and viscoelasticity (Young's modulus = 260 kPa, storage modulus (*E*′) = 35 MPa, loss modulus (*E*″) = 6 MPa; see Figure S3a,b in the Supporting Information), c) low density of 0.1 g cm^−3^ for greater buoyancy and permanent retention at the air–water interface even under mechanical agitation (Figure 1A,B; Movie S1, Supporting Information). Moreover, the PH is also thermally stable up to 300 °C (Figure S3d, Supporting Information). The white color of PH implies its low absorptance (< 20%) and high reflectance (> 60%) over the entire solar spectrum of 250–2500 nm (Figure S3c, Supporting Information); it is mandatory to integrate it with a suitable material for solar–thermal conversion material, that possesses a) broadband absorptance, b) strong photon thermalization, and c) high thermal effusivity. We leverage a combination of morphological tuning and structural engineering to achieve these properties in the solar–thermal convertor.

### 2.2 Design and Structure–Property Correlation of Solar–Thermal Convertor

Accordingly, dendritic fibrous nanosilica (DFNS) was selected as a sacrificial template for the fabrication of NCFs, through chemical vapor deposition using acetylene as a gas‐phase source of carbon to ensure uniform, complete, and conformal infiltration and deposition of carbon within the pores of DFNS.^[^ ^81^ ^]^ This strategy ensured that the morphological advantage of DFNS is completely captured (Figure 1D; Figure S4, Supporting Information). Subsequent removal of DFNS through alkali‐etching achieves NCF with unique open‐ended, floral morphology, graded porosity, and high specific surface area (540 m^2^ g^−1^; Figure 1E) that facilitates multiple reflections accounting for its high broad‐band absorptance of 96% over 250–2500 nm (Figure 1F; Figure S4d, Supporting Information). Such porosity engineered carbons have been utilized for various applications ranging from supercapacitive energy storage, nonplasmonic sensors, supports for electrocatalysts, and exhibit interesting light–matter interactions.^[^ ^80^, ^82^, ^83^, ^84^, ^85^, ^86^, ^87^, ^88^ ^]^

The PH scaffold was transformed into a solar–thermal interfacial evaporator by spray‐depositing a uniform coating of NCF on it, with no identifiable change in its structure or properties (Figure 1A–C; Figure S5, Supporting Information). The large interfacial area provided by NCF (specific surface area, SSA = 540 m^2^ g^−1^ and pore volume 0.7 cc g^−1^; Figure 1E) is expected to facilitate rapid transfer of thermal energy to the interfacial water. The broad (002) reflection in powder X‐ray diffraction pattern and the excitation‐dependent spectral position of the D‐band in the Raman spectra reveal the graphitic ordering dimensions (*L*_c_) as 4–6 nm, while the lateral (*L*_a_) dimensions are 35–50 nm, along with a defect density of 2.65 × 10^11^ cm^−2^ (Figure S6e,f, Supporting Information). Finally, the high resolution transmission electronic microscopy (HR‐TEM) images reaffirm the coexistence of short‐range graphetically ordered domains that show intrinsic disorder among them at the long range (Figure S6a–d, Supporting Information).^[^ ^80^, ^82^, ^83^, ^84^, ^85^, ^86^, ^87^, ^88^ ^]^ Such a hard‐carbon structure synergizes strong photon‐initiated phonon activation over the entire solar spectrum due to the short‐range graphetically ordered domains with the highly restricted phonon propagation due to its long‐range disordered domains. Specifically, the disordered domains serve as scattering points for the phonon vectors (*E* → *k* dispersion domain within the first Brillouin zone.^[^ ^82^ ^]^ Such photon thermalization coupled with the localization of thermal energy results in its low thermal conductivity (1.5 W m^−1^ K^−1^) and high thermal effusance (519 W s^0.5^ m^−2^ K^−1^).^[^ ^89^ ^]^

### 2.3 Synergized Solar–Thermal Interfacial Convertor

Consequently, the integration of PH with NCF through spray coating results in NCF@PH that synergizes the best properties of both these components (Figure 1A–D). NCF@PH was subjected to thermal shock testing, wherein its temperature was repeatedly varied from 120 to 25 °C. The differential scanning calorimetry (DSC)of NCF@PH exhibits no noticeable changes after such thermal stress, pointing to its chemical and structural integrity (Figure S7, Supporting Information). Further, tensile testing (Young's modulus = 260 kPa) and dynamic mechanical analysis of the NCF@PH also confirm its mechanical robustness (Figure S3, Supporting Information). The NCF@PH scaffold also exhibits cohesiveness and mechanical integrity without any signs of delamination in a scotch‐tape peel‐off test (Figure S8, Supporting Information) or fouling when forcibly submerged under water for more than 48 h (Movie S1, Supporting Information). The strong adhesion of NCF to PH is possibly due to the hydrogen bond interactions between the dangling ─SH groups of PH and the ─OH of NCF, as indicated by the 2650 and 3600 cm^−1^ (Figure S2d, Supporting Information). Importantly, this synergistic integration results in strong light absorption by NCF@PH, as seen from the high absorptance (≈ 95%) and low reflectance (<5%) over the broad‐band solar spectrum of 250–2500 nm (Figure 1F).

The advantage of the single‐step spray‐coating approach enables precise tuning of its surface coverage (Figure 1G), leading to loadings ranging from 0.12 to 0.69 mg cm^−2^ that are labeled sequentially as NCF@PH1 to NCF@PH5. While NCF@PH1 (0.12 mg cm^−2^) exhibits nonuniform coating at the nanoscale, high surface coverage (NCF@PH5, 0.60 mg cm^−2^) results in delamination of NCF from PH (Figure S8, Supporting Information). We find that the surface coverage also has a significant effect on the surface temperature (*T*_s_), with NCF@PH3 (0.37 mg cm^−2^) providing the highest *T*_s_ of 120 ± 5 °C (Figure 1G). Thus, NCF@PH3 that provides the highest *T*_s_ and coating stability is subsequently employed in all further experiments. Furthermore, higher loading of NCF (>0.37 mg cm^−2^, NCF@PH4 and NCF@PH5) results in lower *T*_s_ (92 ± 3 and 80 ± 2 °C; Figure 1G). This is due to the creation of additional parasitic channels for thermal dissipation at higher surface coverage and substantially lower contribution from subsurface NCF toward solar absorption and thermal conversion.

The kinetics of the solar–thermal conversion by NCF@PH also warrants further investigation. Accordingly, the temporal evolution of temperature from all the samples reveals that NCF@PH3 and NCF@PH2 possesses similar heating rates (0.08 °C s^−1^), although the cooling rate is higher for NCF@PH3 (0.27 °C s^−1^) compared to NCF@PH2 (0.16 °C s^−1^; Figure 1H). This signifies that NCF@PH3 is not only able to heat faster but also exchanges the thermal energy rapidly with the surroundings. This aspect is further ascertained by its large thermal effusance (519 W s^0.5^ m^−2^ K^−1^) and its uniformly extensive interfacial area. These observations point to the exclusive role of NCF coating in the observed photothermal energy‐conversion process. Furthermore, we establish the NCF@PH3 is effective in minimizing the convective and radiative losses to result in maximum heat transfer to the surroundings and specifically to the interfacial water during the subsequent experiments (vide infra).

### 2.4 Solar–Thermal Interfacial Water Evaporation with NCF@PH

**Figure 2:**
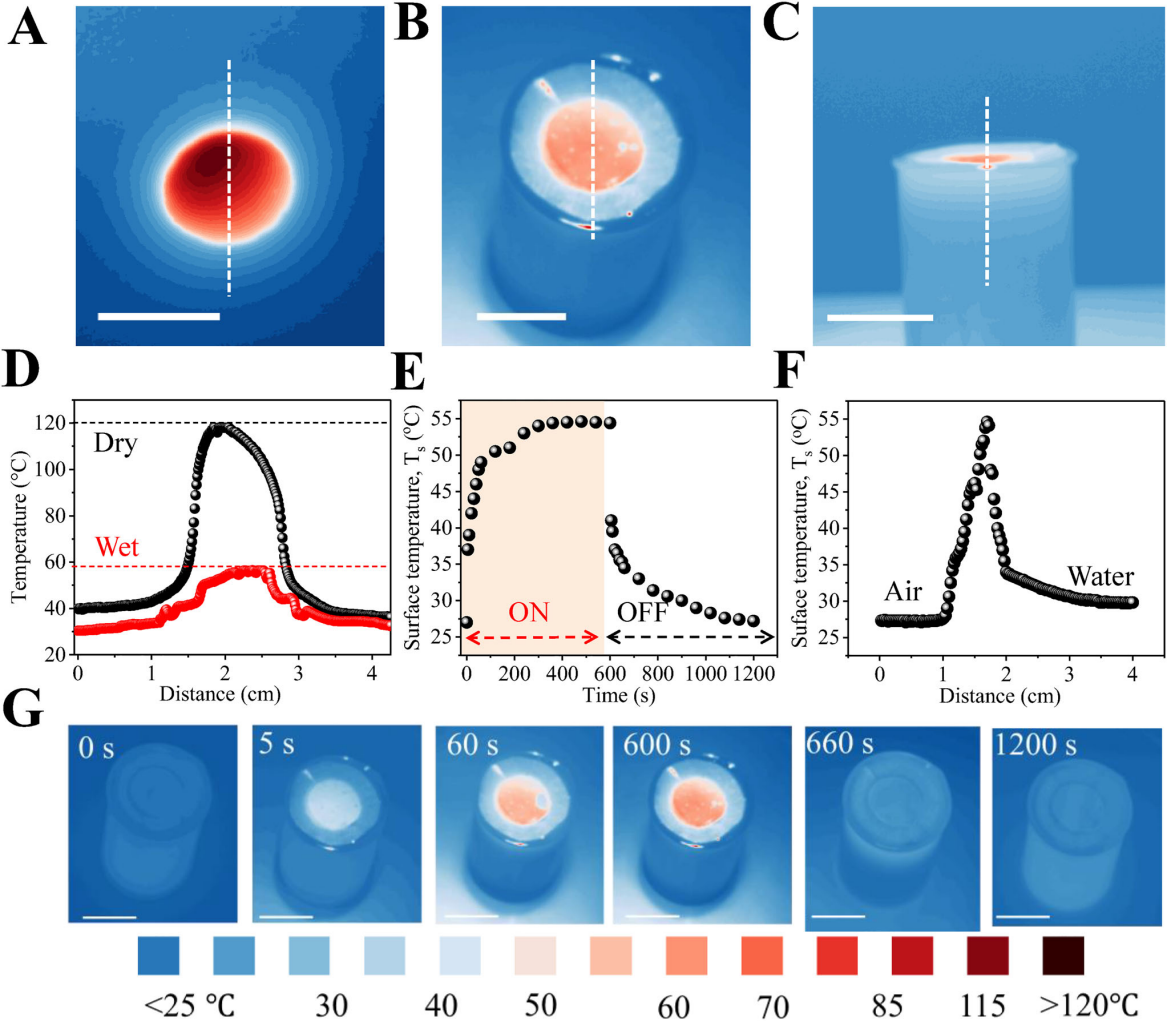
Solar–thermal energy conversion by NCF@PH3. Thermometric images of NCF@PH3 in A) dry condition, and B) at air–water interface in the interfacial configuration. C) The thermometric image of panel (B) captured in side‐way orientation. D) Thermal profile of NCF@PH3 in dry and wet conditions, measured across the dotted lines shown in panels (A) and (B), respectively. E) Temporal variation in *T*_s_ of NCF@PH3 in interfacial evaporator configuration. F) Thermal profile along the dotted line indicated in panel (C). G) A series of thermometric images, recorded in the interfacial configuration during the ON and OFF states of irradiation. All scale bars correspond to 2 cm.

Owing to its low density and porous nature, NCF@PH3 automatically floats on water and self‐assembles to create a tri‐junction between the water film, NCF@PH3, and the incident photons (Figures 1A and 2B; Figure S9, Supporting Information). Herein, the buoyancy of NCF@PH3, coupled with its hierarchically interconnected pore structure, facilitates continuous channels for water transport. The pristine PH side of Janus‐faced NCF@PH3 is oriented toward the water reservoir and exhibits a lower water contact angle (119°) compared to the NCF‐coated surface facing the sunlight (128°, Figure 1I; Figure S10, Supporting Information). Given that both these water contact angles are in the hydrophobic domain, the water transport through NCF@PH3 is mainly guided by the Cassie–Baxter induced capillary wicking, mediated by the presence of surface ─SH groups in pristine PH.^[^ ^90^ ^]^ Subsequently, as the water reaches the NCF coating, where the wettability is not the critical factor, its rapid evaporation is ensured by the efficient solar–thermal energy generation by the NCF coating.^[^ ^89^ ^]^ Furthermore, this trend of contact angle is uniformly observed over a wide range of salinities (0–35 000 ppm), although higher salinity leads to lowering of contact angle due to the Coulombic interactions between the ─SH and the solvated ions (Figure 1I).

Thermometric imaging, under solar illumination, of NCF@PH3 exhibits a clear and uniform increase in the *T*_s_ from room temperature 25 to 120 °C within 200 s (**Figure** **2**A,D; Figure S11, Supporting Information). Importantly, the *T*_s_ for NCF@PH3 is substantially higher than its immediate ambience, indicating a positive effect of thermal energy generation by NCF. The thermal profile of the NCF@PH3 in both the dry and wet conditions exhibits identical profiles, although the *T*_s_ is substantially higher in the dry state than the wet state (Figure 2B,D). Thus, the temperature difference produced between the dry and wet NCF@PH3 is ≈60 °C and provides a measure of the usable heat energy transferred to the interfacial water for its heating and evaporation (Figure 2A,B,D).

In the interfacial configuration, the hydrophobic nature of the interfacial evaporator ensures that the NCF@PH3 is always localized at the air–water interface, exhibiting a *T*_s_ of 54 ± 1.5 °C (Figure 2B,C,E,G). Importantly, the time taken to reach this *T*_s_ is similar for both dry and wet states (200 and 250 s, respectively; Figures 1H and 2E), indicating the rapid equilibration achieved with the ambience through NCF@PH3. Herein, the localized heating produced by the NCF coverage along with the extremely low thermal conductivity of the PH membrane minimizes the parasitic convective heat‐transfer pathways. Furthermore, a rapid decrease in the *T*_s_ is observed after switching off the illumination (Figure 2E,G). The similarity in heating rate (0.4 °C s^−1^) and cooling rate (0.3 °C s^−1^) of NCF@PH3 at the air–water interface indicates the continuous convective flow of thermal energy to the interfacial water film. Even after prolonged light exposure (120 min), the temperature of the air immediately above the surface of NCF@PH3 remains at 27 °C, while the bulk water beneath it maintains a temperature of 30 °C (Figure 2C,F). This reaffirms the minimal heat loss from the NCF@PH3 to both the ambient air and bulk water and stems from the effective thermal insulation provided by the uncoated PH facing the water reservoir that cuts off any convective heating of bulk water. Thus, effective and rapid channelizing of the solar–thermal energy to the interfacial water is promoted by the Janus geometry of the NCF@PH3. Thereby, the NCF@PH3 interfacial evaporator eliminates the need for any additional support to a) maintain its buoyancy in water and b) for thermal blocking of bulk heating, thereby converging two important requirements in a single material.^[^ ^70^ ^]^ In fact, the Janus structure developed here is also the thinnest interfacial evaporator reported thus far (Figure S12, Supporting Information).^[^ ^50^, ^62^, ^65^, ^91^, ^92^, ^93^, ^94^ ^]^

#### 2.4.1 Confined Solar–Thermal Heating by NCF@PH

**Figure 3:**
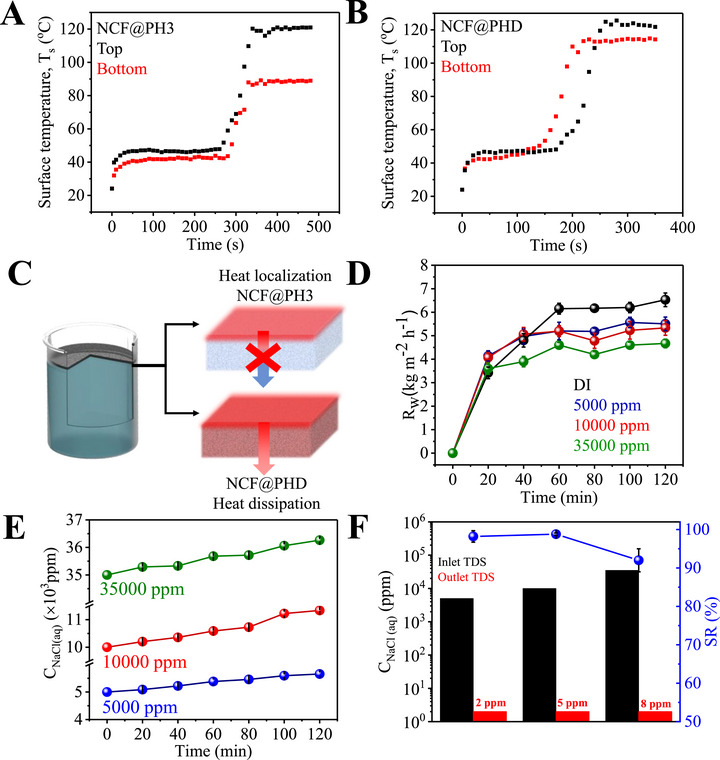
Effect of Janus geometry in localized heating. A) Temporal variation of *T*_s_ on the NCF‐coated (top) and uncoated (bottom) surfaces of Janus‐faced NCF@PH3. B) Similar temporal variation in *T*_s_ from the top and bottom surfaces of NCF@PH containing NCF on both the top and bottom surfaces of PH, termed as NCF@PHD. C) Schematic representation of localized interfacial heating achieved with Janus‐faced NCF@PH3, while NCF@PHD shows heat dissipation leading to lower *T*_s_, as observed in panel (B). D) Kinetics of water evaporation (*R*_w_) with water feedstock of varying salinities (0–35k ppm). E) Change in salinity of the water feedstock during the evaporation experiments in panel (D). F) Salinity of the solar thermally distilled water, compared with that of water feedstock, along with the estimated salt rejection (%) at these varying salinities ranging from 0 to 35k ppm (Table S4, Supporting Information).

Considering that the porosity of PH extends continuously throughout the thickness of the membrane, it is important to understand the effect of localized heating on the kinetics of heat transfer and water evaporation (**Figure** **3**). Accordingly, an isolated drop of water (200 µL) is placed on a suspended NCF@PH3 membrane that is monitored by a thermal camera from both its Janus faces. These Janus faces are referred to as top side for the NCF‐coated face and bottom side for the pristine PH face. The capillarity of the membrane ensured uniform penetration and retention of the water drop across its thickness. The initial dark temperatures of both the top and bottom sides are identical.

Upon solar illumination, a rapid increase in the temperature of the top side is observed within the first 25 s, while its bottom side reaches a noticeably lower temperature within the same time (Figure 3A). Thus, a temperature difference of ≈10 °C is produced between the top and bottom sides of the NCF@PH3 membrane (Figure 3A) and is attributed to the presence of water, that serves to convectively equilibrate the thermal energy generated by the NCF coating. However, no such thermal equilibrium is observed between the top and bottom sides, even after a prolonged time interval (250 s), indicating the effective blocking of thermal transport from the top to bottom sides of NCF@PH3. Consequently, the complete evaporation of the water drop is achieved in ≈300 s, resulting in a mass‐transfer limited *R*_w_ of 1.2 kg m^−2^ h^−1^ for NCF@PH3. The evaporation was confirmed visually and also by the sharp increase in the *T*_s_ of the top side to 120 °C (Figure 3A). Both the *T*_s_ and the kinetics of heating are in good agreement with that observed for dry NCF@PH3 (Figure 1H). Finally, while the bottom side saturates at *T*_s_ of 90 °C, the NCF‐coated top side reaches a significantly higher temperature of 120 °C (Figure 3A). This reaffirms the thermal blocking nature of anisotropic NCF@PH3.

To ascertain these observations, control experiments are repeated with the suspended PH membrane that is uniformly coated with NCF from both the top and bottom sides, and therefore referred to as NCF@PHD. In this configuration, in addition to the water drop, the continuous NCF coating is also expected to contribute to heat dissipation. Accordingly, in the presence of water drop, the temperatures of the top and bottom surfaces are similar (47 and 45 °C, respectively) although the solar illumination is done only on the top side (Figure 3B). This confirms rapid and effective heat transfer across the thickness of the NCF@PHD membrane. Again, the *T*_s_ values of both top and bottom sides of NCF@PHD reach similar temperatures (120 °C) after all the water is evaporated, indicating a strong heat‐dissipation channel due to the continuous NCF coating in NCF@PHD (Figure 3B). The mass‐transfer limited *R*_w_ for this configuration is estimated as ≈1.8 kg m^−2^ h^−1^, that is higher than that of NCF@PH3 (1.2 kg m^−2^ h^−1^). Again, the effective delocalization of the thermal energy on both sides of the NCF@PHD membrane causes faster evaporation of the water from all sides of NCF@PHD, accounting for the increased *R*_w_. However, it is to be noted that NCF@PHD would effectively leak the thermal energy to the bulk water in an interfacial evaporator configuration and therefore would be ineffective compared to the anisotropic, Janus‐faced NCF@PH3 (Figure 3C). Thus, we achieve the effective channelizing of the solar–thermal energy to interfacial water in NCF@PH3, that is captured in the schematic (Figure 3C). Such unique features of Janus‐faced, ultrathin, and unsinkable NCF@PH3 represent strong advantages over several other systems demonstrated in literature in two important aspects.

- First, the additional interfaces created by employing extra thermal blockers beneath the interfacial evaporator lead to substantially longer water‐transport channels, impeding *R*_w_.
- Heat dissipation to the bulk water leads to both lower *R*_w_ and promotes the formation of air gaps within the water‐transport channels.

These important limitations are eliminated with thin and unsinkable NCF@PH3. In fact, the NCF@PH3 is one of the thinnest interfacial evaporators when compared to several other materials (Figure S11, Supporting Information).

#### 2.4.2 Performance of NCF@PH with Saline Water

Accordingly, experiments in interfacial evaporative configuration with NCF@PH3 yielded significantly higher *R*_w_ values of 6.5, 5.5, 5.3, and 4.6 kg m^−2^ h^−1^ for pure water, 5000, 10 000, and 35 000 ppm (85, 170, and 595 mm of NaCl), respectively (Figure 3D; Figure S13, Supporting Information). As expected, the *R*_w_ is significantly improved from a mass‐transfer limited value (1.2 kg m^−2^ h^−1^, Figure 3A) in all these cases and also corresponds to one of the fastest evaporation rates achieved thus far (Figure 3D; Figure S13, Supporting Information). The slight decrease in *R*_w_ for saline water originates from a colligative effect. Interestingly, the similarity in *R*_w_ in the case of 5000 and 35 000 ppm (Figure 3D; Figure S13, Supporting Information), in spite of the sevenfold higher salinity for the latter, indicates the minimum influence of salinity on the performance of NCF@PH3 and warrants further investigation.

Accordingly, the salinity in the water reservoir was continuously monitored during the water evaporation process and found to exhibit a linear, continuous, and monotonic increases from its starting value for the entire duration of the experiment (120 min, Figure 3E). Interestingly, starting from the feedstock salinities of 5000, 10 000, and 35 000 ppm, the salinities of all the water feedstocks increased to 5653, 11 336, and 37 783 ppm, respectively (Figure 3E). Furthermore, the relative increase in salinity of the reservoir was constant at 0.13, irrespective of the salinity of the feedstock and strongly reiterates the similar *R*_w_ observed for the entire range of salinities tested (Figure 3D,E). The knowledge of *R*_w_ and the increased salinity of the water reservoir enables us to theoretically assess the salinity expected in the reservoir corresponding to 100% SR by NCF@PH3 (see Tables S1 and S4, and Section S1.1 in the Supporting Information). Such a scenario would imply the complete removal of salt from the NCF@PH3 through its dissolution into the water reservoir and, therefore, would result in feedstock salinities increasing to 5760, 11 457, and 39 772 ppm (Section S1.1 in the Supporting Information). Given that the experimentally observed salinities in the reservoir are consistently lower than the theoretical estimates, the actual SR from the NCF@PH3 was estimated as 98.5% up to 10 000 ppm and a marginally lower 92% for 35 000 ppm (Figure 3F). Again, the lowering of SR was ≈6.5% in spite of enhancing the feedstock salinity by 3.5 times (from 10 000 to 350 000 ppm) and indicates the robustness and reliability of performance from NCF@PH3 (Figure 3F). These observations are also in excellent agreement with the weight gain observed in NCF@PH3 after the experiments, validating the performance. Given that water evaporation proceeds uniformly from the entire NCF@PH3 surface, the residual salt deposition occurs at surface coverages of 58.8, 64, and 322 µg cm^−2^ h^−1^ and over the NCF@PH3 evaporator, corresponding to the three salinities tested (5000, 10 000, and 35 000 ppm). Assuming that this performance is retained over longer duration, which is validated in subsequent discussions (vide infra), we estimate a theoretical continuous working potential of 603 h (≈3300 kg m^−2^ water evaporation), 565 h (≈2900 kg m^−2^ water evaporation), and 55.4 h (≈270 kg m^−2^ water evaporation) with 5000, 10 000, and 35 000 ppm feedstocks, respectively (Table S2 and Section S1.2 in the Supporting Information). These results have encouraged us to carry out field testing of this system in Kutch, Gujarat, to validate these long‐term predictions.

### 2.5 Understanding Salt Rejection and Stability of NCF@PH3

**Figure 4:**
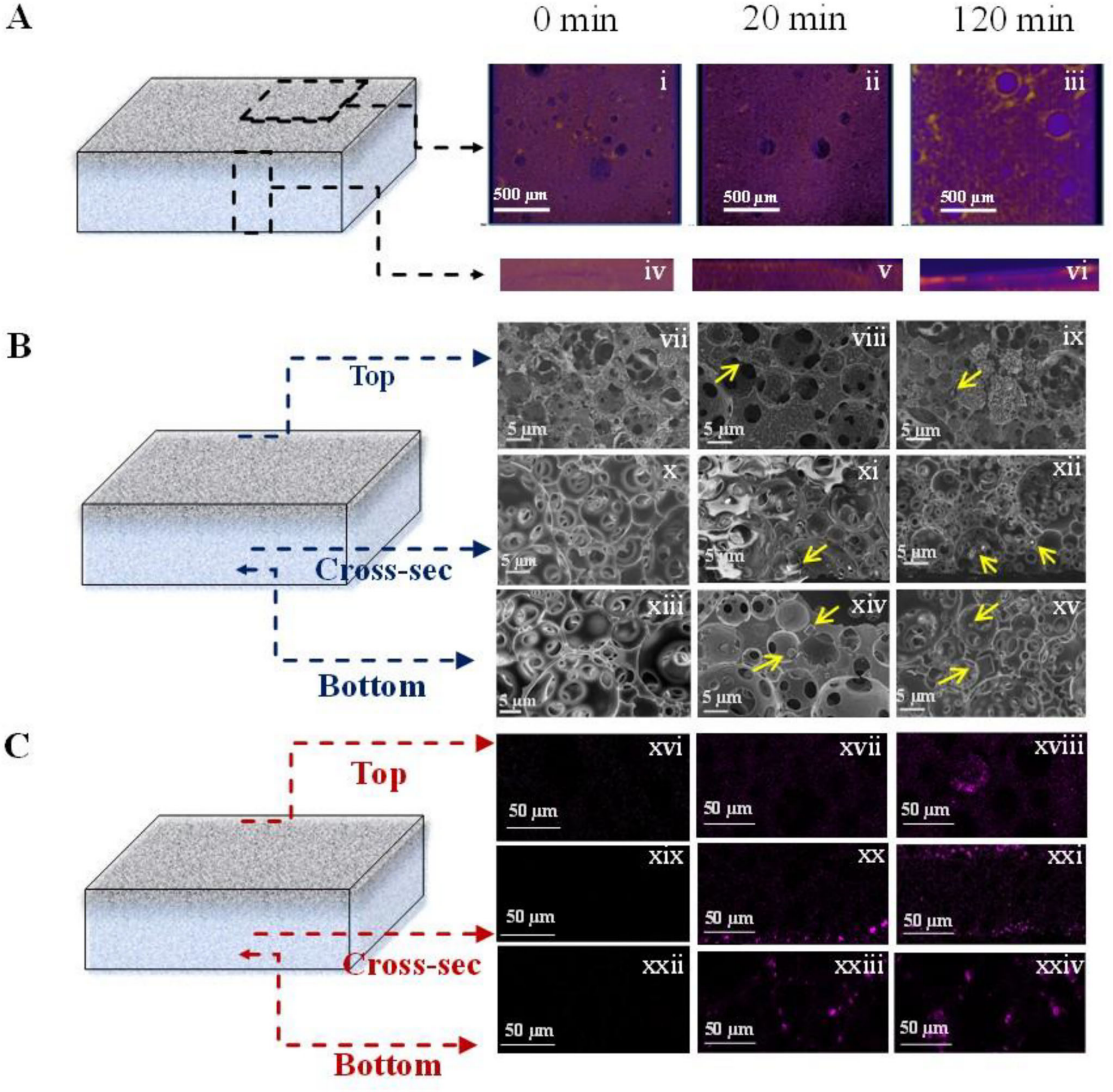
Microscopic understanding of water transport and salt deposition in NCF@PH3. A) 3D X‐ray tomography of i–iii) top and iv–vi) cross‐sectional surfaces of NCF@PH3, carried out at different time instances of solar‐water evaporation with 5000 ppm saline water feedstock. B) SEM images of the same samples and time periods (0, 20, and 120 min) recorded on vii–ix) top, x–xii) cross section, and xiii–xv) bottom sections of NCF@PH3. C) Energy dispersive X‐ray spectroscopy (EDS) elemental mapping, based on Na, for the same samples recorded on xvi–xviii) top, xix–xxi) cross section, and xxii–xxiv) bottom sections of NCF@PH3.

Such a superior salt‐rejection rate from NCF@PH3 can originate due to the chemical hydrophobicity of both the PH and NCF@PH surfaces (110° and 120°, Figure 1I), which makes salt depositions unfavorable.^[^ ^95^ ^]^ This was probed using a range of microscopic techniques that offer varying spatial resolutions. First, 3D X‐ray tomographic sections of the used NCF@PH3 membranes were done to probe the salt deposition along two domains:

1. Top, NCF‐coated, water‐evaporating surface (**Figure** **4**A(i–iii))
2. Cross section across which water transport occurs (Figure 4A(iv–vi)).

Given the higher atomic number of Na compared to C of NCF and PH, any deposition of salt on these two areas is expected to provide a clear contrast in the 3D X‐ray scans. Thus, pristine NCF@PH3 presented a uniform surface and cross section, with lower intensity domains coming from the pores of PH (porosity ≈ 80%). Further, these images reveal no observable “bright‐spots” or high‐intensity regions during the first 20 min of water evaporation (5000 ppm). Upon continuing the water evaporation for 120 min, high‐intensity, bright spots were observed on both the surface and cross sections of the NCF@PH3 (Figure 4A(iii,vi)).

Such spots arise from deposition of NaCl and are primarily confined to the boundaries of the porous channels on the surface of NCF@PH3. Simultaneously, the cross‐sectional images also show accumulation of salt on both the top and bottom surfaces of the NCF@PH3 membrane. However, there was no intense spot in the middle of the membrane, indicating the possibility of lesser salt deposition along the channels connecting the top and bottom surfaces of the Janus‐faced NCF@PH3. This is an important observation that essentially points to the free, unrestricted movement of water from the bulk to the evaporating interface, along the channels of NCF@PH3. However, the spatial resolution and chemical sensitivity of the technique do not enable us to confirm these observations. Therefore, we carried out identical investigations using SEM coupled with Na‐based elemental mapping (Figure 4B(vii–xv),C(xvi–xxiv)). These images exhibit clear evidence of salt deposition exclusively on the bottom side of NCF@PH3, that is in contact with the saline water, after 20 min of evaporation (Figure 4B(viii,xi,xiv),C(xvii,xx,xxiii)). However, no salt deposition is observed in the cross‐sectional and the evaporating surfaces of the same sample. Subsequently, after 120 min of the experiment, prominent salt‐deposited domains were observed on both the evaporating and bottom surfaces of NCF@PH3, while the middle portions remained salt free (Figure 4B(ix,xii,xv),C(xviii,xxi,xxiv)). Thus, the salt deposition was exclusively confined to the boundaries of the pores on the top and bottom surfaces, with no detectable salt present between the channels connecting these two surfaces. Thus, these results validate that the channels of NCF@PH3 remain salt free and allow continuous water transport through them. Therefore, the hydrophobic and porous nature of the PH scaffold functions as a self‐cleaning surface by morphologically facilitating water transport while simultaneously impeding salt deposition within its channels—conditions that are critical for long‐term usability and durability of the interfacial evaporator.^[^ ^25^ ^]^

### 2.6 Benchmarking NCF@PH and Its Versatility

Based on the above discussions, the solar–thermal conversion efficiency (*η*‐STC) and solar‐vapor conversion efficiency (*η*‐SVC) are estimated as follows

(1) $$\left(\eta \mathrm{STC}\right)=\frac{\mathrm{Pevaporation}}{\mathrm{Plight}}\times 100$$

where *P*_light_ refers to the incident solar power, *P*_evaporation_ is the effective power used by evaporation, after accounting for radiation and convection (Section S1.3 in the Supporting Information)

(2) $$\left(\eta SVC\right)=\frac{R_{w}\;\left(\Delta H_{\mathrm{LV}}+Q\right)}{E_{\mathrm{light}}}\times 100$$

where *R*_w_, *∆H*_LV_, *Q*, *m*, *c*, *∆T*, and *E*_light_ refer to the water‐evaporation rate, latent heat of evaporation of water, heat delivered to interfacial water, mass of the evaporated water, specific heat capacity of water, temperature difference achieved with NCF@PH3 and solar‐energy density, respectively (Section S1.3 in the Supporting Information).

**Figure 5:**
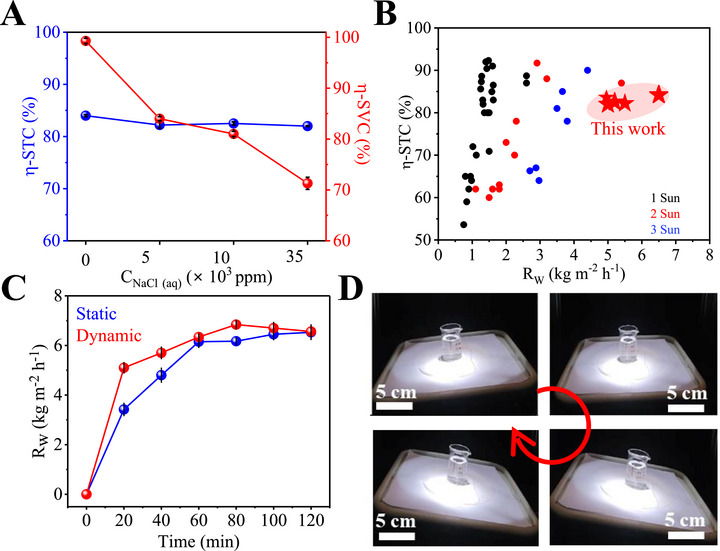
Benchmarking performance and versatility of NCF@PH3. A) Variation in solar–thermal conversion efficiency (*η*‐STC) and solar‐vapor conversion efficiency (*η*‐SVC) of NCF@PH3 with deionized (DI) water and saline solutions (5000–35 000 ppm). B) Ashby plot comparing the performance of NCF@PH3 (red star) with 43 other similar reports from literature, establishing the superior performance of NCF@PH3 with respect to *η*‐STC and *R*_w_.^[^ ^36^, ^93^, ^96^, ^97^, ^98^, ^99^, ^100^, ^101^, ^102^, ^103^, ^104^, ^105^ ^]^ C) Assessing the performance of NCF@PH3 under static conditions, without any agitation to the water, and in dynamic conditions, where the water feedstock is undergoing a sinusoidal oscillation at a frequency (0.5 Hz) comparable to tidal waves. D) Optical photographs of the evaporation setup under various stages of such dynamic turbulence.

Thus, the estimated *η*‐STC ranges from 84% to 82% (0–35 000 ppm) with the corresponding *η*‐SVC of 99–71% (**Figure** **5**A; Table S3, Supporting Information). Further, the effect of such high salinities on the enthalpy of evaporation was also estimated.^[^ ^66^, ^106^ ^]^ In spite of marginally lower *H*_LV_ for saline water, the decrease in the *R*_w_ contributed to the overall lowering of the *η*‐SVC (Table S8, Supporting Information). This clearly indicates that the performance of NCF remains stable and invariant even in extreme saline environments, while the *η*‐SVC drops marginally in the saline environments, due to the salt deposition. Such high performance warrants an exhaustive comparison of NCF@PH with respect to several other reports from the literature that describe planar 2D evaporators, in order to benchmark and assess its potential for sustainable interfacial water evaporation (Figure 5B). Thus, the Ashby plot of *η*‐STC versus *R*_w_ (Figure 5B) establishes NCF@PH3 as an unique solitary material that overcomes the fundamental tradeoff between *η*‐STC and *R*_w_ to deliver outstanding performance of 84% and 6.5 kg m^−2^ h^−1^, respectively, in a vast domain of other materials, including vertically alligned carbon nanotubes (VACNTs), functionalized reduced graphene oxide  (f‐RGO), TiO*_x_*, c‐mushroom, and plasmonic materials (Figure 5B; Table S6, Supporting Information).^[^ ^43^, ^51^, ^91^, ^97^, ^98^, ^99^, ^104^, ^105^, ^107^, ^108^ ^]^ Notably, few materials such as vertically aligned graphene and graphene oxide–polyethylene glycol (PEG) exhibit *η*‐STC greater than NCF@PH3; the *R*_w_ is highest for NCF@PH3 (red stars) across the wide range of salinities tested (0–35 000 ppm). Thus, NCF@PH3 occupies a unique, solitary position in the Ashby plot with excellent performance on both *η*‐STC and *R*_w_ (Figure 5B). Overcoming this fundamental tradeoff between *η*‐STC and *R*_w_ to deliver a superior performance originates from the synergistic combination of several engineered characteristics of NCF@PH3 (vide supra) as follows:

1. Janus‐faced NCF@PH3 achieving heat localization and effective transfer to the interfacial water, with minimal loss to bulk water;
2. Continuous, interconnected pore structure that facilitates water transport through structure‐induced capillarity to ensure a constant supply of water to the interfacial surface and thereby minimize mass‐transfer limitations,
3. Hydrophobic surface impending salt deposition within its channels,
4. High broad‐band solar absorptance and structurally engineered structure of NCF that promotes photon thermalization with greater effusivity.

Leveraging the unsinkable and stable nature of NCF@PH3, we demonstrate invariant performance of the *R*_w_ under the influence of perturbation that mimics the wave movement in oceans.^[^ ^109^ ^]^ Experiments were carried out by applying a sinusoidal wave frequency of 0.5 Hz (typical wave frequency in oceans ≈ 0.14 Hz) to test the efficacy of NCF@PH3 (Figure 5C,D; Movie S2, Supporting Information). Again, the *R*_w_ of such a dynamically perturbed system is invariant and identical to the static system and indicates that NCF@PH3 is always present at the water–air interface without submerging (Figure 5D). Interestingly, the interfacial evaporator adjusts to the continuously varying angle of incidence (from −10° to 10°) and exhibits invariant performance due to the strong isotropic, angle‐independent absorption of NCF. Finally, the ambient and bulk‐water temperatures are identical during such continuous motion, with the NCF@PH3 being selectively heating to 54 ± 3 °C (Movie S2, Supporting Information).

### 2.7 Correlating Water Structure and Energetics with *R*_w_

**Figure 6:**
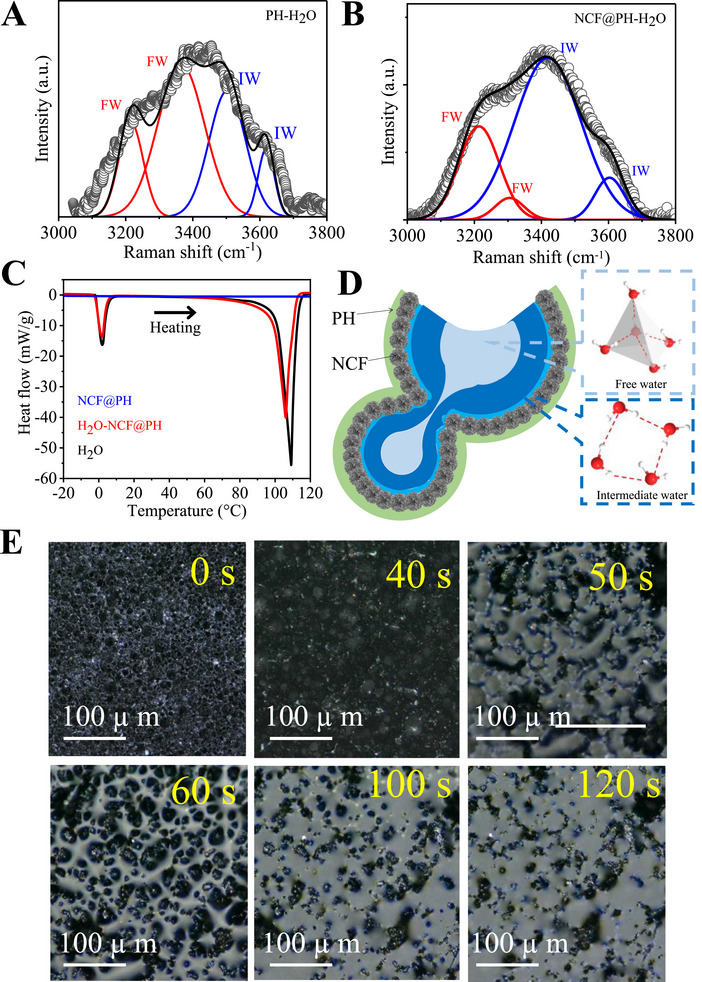
Spectroscopic analysis of different states of water in NCF@PH3. Raman spectra of A) PH–H_2_O and B) NCF@PH–H_2_O in the region from 3200 to 3600 cm^−1^. The deconvoluted spectra are assigned to free water (FW) and intermediate water (IW) based on the Raman shifts at 3213 and 3307 cm^−1^, and 3420 and 3602 cm^−1^, respectively. C) DSC profile bulk water and water contained within NCF@PH3, for evaluating the latent heat of vaporization. Pristine NCF@PH3 does not show any phase transition in the temperature range (from −20 to +120 °C) probed. D) Schematic representation of the various states of water in the cross section of NCF@PH3, showing interconnected and continuous IW and FW domains, spread along the walls of NCF@PH3 and within the bulk pores, respectively. E) Time‐lapse optical microscopic images showing the capillary wicking of water by NCF@PH3.

In addition to all the above experimental evidence, the water evaporation process is strongly dependent on the state of water within the micro‐ and mesoporous channels of PH and NCF@PH3. We proceed to probe the different states of water held within the NCF@PH3 using micro‐Raman spectroscopy (**Figure** **6**).^[^ ^60^, ^110^, ^111^ ^]^ The Raman spectrum of water exhibits the characteristic asymmetric and symmetric stretching modes at ≈3230 and ≈3500 cm^−1^ in the case of both PH and NCF‐PH (Figure 6A,B; Figure S15, Supporting Information). It is to be noted that this spectral region corresponds exclusively to water without any interference from PH or NCF (Figure S15, Supporting Information). Therefore, deconvolution of this band enables us to understand the nature of water that is contained within both the pristine PH and NCF@PH3. Accordingly, the spectra were fitted with four Gaussian profiles for direct quantitative comparison of the hydrogen‐bonded network existing in the water confined within both these membranes (PH–H_2_O and NCF@PH3–H_2_O) (Figure 6A,B). The spectra of NCF@PH3–H_2_O show Raman peaks at 3215 and 3300 cm^−1^ corresponding to the water molecules present in the ideal tetrahedral H‐bonded network (labeled as free water, FW, Figure 6D). In addition, the peaks at 3407 and 3600 cm^−1^ correspond to the intermediary water molecules that contain fewer H‐bonds and therefore weakly interact with each other (IW, Figure 6D). Accordingly, the ratio of IW/FW provides a quantifiable estimate of the water molecules that are loosely bound and, therefore, is expected to require lower energy for evaporation. The IW/FW ratio increases from 0.77 for pristine PH to 1.25 for NCF@PH3. The proportion of such loosely bound IW shows an increase from 46% in the case of pure PH to 64% for NCF@PH3, indicating substantial activation of water molecules within the pores of NCF@PH3, leading to higher *R*_w_. While the surface ─SH functionalities in pristine PH creates a strong and tightly packed domain of bound water, the large macroporous channels enable the containment of greater proportion of FW in pristine PH. In contrast, NCF@PH disrupts the bound water domain due to the continuously graded pore structure (micro‐ to mesopores) of NCF, besides constricting the macroporous channels of PH. Therefore, the corrugated NCF coating leads to an increase in IW state, leading to lowered energy required for water evaporation and higher *R*_w_. Based on several earlier reports, the formation of IW and FW would carry distinctly different signatures in the heat‐flow patterns in DSC (Figure 6C).^[^ ^112^ ^]^ Accordingly, the enthalpy of evaporation (∆*H*_LV_), estimated from the DSC profile (Figure 6C), is substantially lower for water confined with NCF@PH3, compared to pure water (Table S7, Supporting Information). However, we acknowledge that the lowering of *∆H*_LV_ is a subject matter of intense debate and merits deeper investigations.^[^ ^113^ ^]^ Recent studies also postulate greater contribution from the meniscus effect for explaining the higher *R*_w_, while negating the differential enthalpy arguments.^[^ ^114^ ^]^ Given that the surface of NCF@PH3 is also roughened and porosity tuned to support the rapid formation of micromenisci at the air–water–light interface (5 µm s^−1^, Figure 6E(i–vi). Moreover, this is also substantiated by the high rate of wicking of water through these channels, as observed through optical microscopic images and Movie S3 (Supporting Information). We observe that the water transports at the rate of 5 µm s^−1^ through the thin interfacial evaporator, leading to the initial formation of a meniscus that subsequently interconnects to result in a thin water film at the surface of the evaporator. Experiments conducted with pristine PH as an interfacial evaporator yields similar evaporation rate as that observed in conventional solar stills (1.5 kg m^−2^ h^−1^). This indicates that the menisci effect contributes a lower extent (23%) to the observed *R*_w_ with NCF@PH, while the dominant contribution arises from the formation of extensive intermediate water leading to lowered evaporation enthalpy. However, given the high rate of water transport through the pores of NCF@PH (5 µm s^−1^), the micromenisci play a critical role in continuously replenishing the water level at the interfacial evaporator by maintaining a constant flow of water to the interface. Therefore, deconvoluting the exact quantitative contributions from these two processes requires advanced measurements and simulations that present distinct opportunities for further studies.^[^ ^115^ ^]^

### 2.8 Heat‐Blocking Mechanism of NCF@PH

The localization of the solar–thermal energy at the evaporating interface of NCF@PH forms an important aspect of this work that is critical for achieving high *R*_w_. Accordingly, this was further investigated through both experiments and numerical modeling. Experimentally, the NCF‐coated side of Janus NCF@PH (80% porosity, 2.0 cm diameter, and 0.8 cm thickness) was exposed to light through a solar simulator (Xe lamp, 300 W), while the temperatures of both the coated and the uncoated surfaces were estimated using thermometric imaging (FLIR A6703c). These temperatures were validated by separate measurements under identical conditions using thermocouples. The temperature gradient established across the thickness of NCF@PH was used to arrive at the thermal conductivity (*K*) as per Fourier's law, given by (Equation (3))^[^ ^116^ ^]^

(3) $$Q=k.\frac{dT}{dx}$$

where *Q* refers to the heat input, that is estimated from the solar–thermal conversion of the input light energy by the NCF coating and d*T*/d*x* refers to the thermal gradient established across the thickness of NCF@PH. Thereby the thermal conductivity, *k*, was estimated at two different solar powers to be 0.12–0.14 W m^−1^ K^−1^ (**Table** **1**).

**Table 1:** Summary of values of thermal conductivity measurement using Fourier's law of heat conduction.

Solar power [W m^−2^]	Corrected heat flux[W m^−2^]	Experimental d*T*/d*x* [K m^−1^]	Experimental thermal conductivity [W m^−1^ K^−1^]
1000	600	4125	0.14
2000	1200	10 000	0.12

Thus, the thermal conductivity of Janus‐faced NCF@PH is one order of magnitude higher than air (0.02 W m^−1^ K^−1^), and less than water (0.60 W m^−1^ K^−1^). These experiments and the estimated values of thermal conductivity indicate a high thermal resistance for the heat flow across the NCF@PH and thereby reinforce the proposed heat‐blocking mechanism. Finally, the bulk water temperature was found to increase by 5 °C during the course of the entire measurement lasting for 10 h, reaffirming the heat localization by NCF@PH (Figure S14, Supporting Information). However, the localization of the heat that would eventually lead to heat blocking is not evident from such experiments and therefore demand numerical modeling.

**Figure 7:**
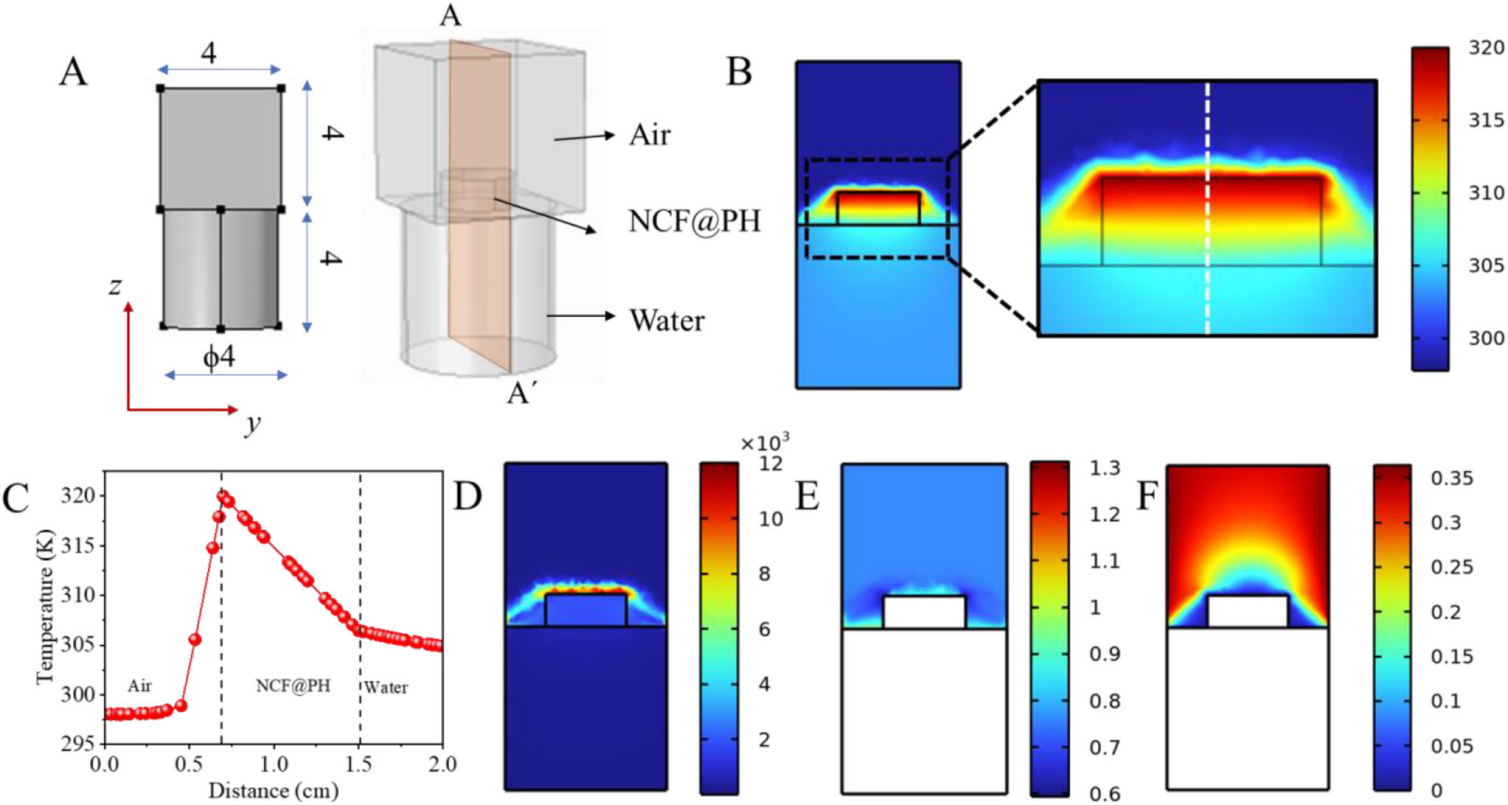
Heat localization at the evaporating interface of NCF@PH. A) A 3D geometrical model of the NCF@PH evaporation setup, giving labels and dimensions of each component. Images of the model depicting B) temperature distribution across the NCF@PH at the air–water interface (color legend in K). C) Spatial variation of temperature estimated across the white line shown in panel (B), signifying the large thermal gradient operating at the interface. D) The temperature difference operating within the domain, with the reference temperature being 25 °C. E) Relative humidity variation, and F) velocity vector imaging of convective air in the air domain.

A numerical model is developed for simulating the evaporation of water through a porous medium floated on the surface of water (COMSOL Multiphysics software). The bottom surface of the water‐saturated porous medium is in contact with the bulk water having a temperature of 30 °C. During the solar illumination, the temperature of the top, porous surface was measured as 47 °C, under steady‐state conditions with ambient temperature and relative humidity (RH) maintained at 25 °C and 50%, respectively. A 3D geometry consisting of an air‐ and water‐saturated domain is considered in this analysis to evaluate the evaporation rate of water from the top surface under the steady‐state condition. The air domain is made sufficiently large to mimic ambient conditions. The water domain is shaped as a cylinder with a diameter and height of 4 cm each, as shown in **Figure** **7**A.

In this numerical model, the following assumptions were made:

- Laminar flow for air with a constant water level throughout the study,
- Newtonian behavior of the fluids,
- Effects of micromenisci at the evaporating surface, due to the porous nature of the NCF@PH, are not considered,
- Vertical, bottom, and side walls of the water domain are perfectly impermeable.

The governing equation for laminar flow in moist air, under these conditions, is given as

(4) $$\rho \left(u.\nabla \right)u=\nabla .\left[-pI+\tau \right]+F$$

where *ρ* is the density, *u* is the velocity, *p* is the pressure, *F* is the volume force vector, and τ is the viscous stress tensor.

The energy equation for the water‐saturated porous medium, considering the thermal equilibrium model, is given by

(5) $${\left(\rho C_{p,}\right)}_{\mathrm{eff}}u.\nabla .q=Q$$

(6) $$q=-k_{\mathrm{eff}}\nabla T$$

(7) $$\begin{array}{cc} & k_{\mathrm{eff}}=\in_{p}\;k_{f}+\left(1-\in_{p}\right)k_{s};\;{\left(\rho C_{p}\right)}_{\mathrm{eff}}\\ & \;=\in_{p}\;{\left(\rho C_{p}\right)}_{f}+\left(1-\in_{p}\right){\left(\rho C_{p}\right)}_{s} \end{array}$$

where *k*_eff_ is the effective thermal conductivity, (*ρC*_p_)_eff_ is the effective volumetric heat capacity at constant pressure, *q* is the conductive heat flux, *u* is the Darcy velocity, *Q* is the heat source, which is set as zero, and *ϵ*_p_ is the porosity.

The moisture transport physics is mainly governed by vapor concentration and is represented by

(8) $$M_{v}\frac{\partial c_{v}}{\partial t}+M_{v}u.\nabla c_{v}+\nabla .g_{w}=G$$

(9) $$g_{w}=-M_{v}D\nabla c_{v}$$

where *M*_v_ is the molar mass of water vapor and *c*_v_ is the molar vapor concentration. *D* is the vapor diffusion coefficient in air (2.6 × 10^−5^ m^2^ s^−1^), *u* is the air velocity field, *g*_w_ is the vapor diffusive flux, and *G* is the moisture source.

#### 2.8.1. Boundary Conditions

2.8.1

For evaporation modeling, the surface of the porous medium is provided with a wet surface boundary condition. The mass of water evaporated is based on the following equation below

(10) $$g_{\mathrm{evap}}=M_{v}\;K\left(c_{\mathrm{sat}}-c_{v}\right)$$

where *M*_v_ is the molar mass of water vapor, and *c* is the molar vapor concentration.

The evaporation rate factor, *K*, is evaluated using Sherwood number correlations^[^ ^117^ ^]^ as given here

(11) $$\mathrm{Sh}=0.230Sc^{\frac{1}{3\;}}Ra^{0.321};\;9.6\times 10^{5}<\mathrm{Ra}<5.7\times 10^{8}$$

where Sh is the Sherwood number, Sc is the Schmidt number, Ra is the Rayleigh number, *D*_c_ is the diffusion coefficient of water vapor in air, and *D* is the diameter of the cylinder.

An open‐boundary condition has been provided at the external surfaces of the air cavity with a temperature of 25 °C. Note that in the experimental study, we have used solar illumination, which radiates a constant heat flux. Experimentally, the top surface temperature is measured to be 47 °C under steady state, which is a result of the incoming radiation, absorption, and emission from the surface, including heat‐loss pathways.

Figure 7F depicts the velocity profile in the plane passing through the center of the plane AA′ (Figure 7A). The velocity profile of the moist‐air domain shows that velocity increases with the distance away from the surface due to rise of hot air because of natural convection. Water vapor concentration gradient can be observed from the relative humidity plot (Figure 7E). The evaporation is mainly driven by diffusion near the top surface. It is observed that the RH at the top surface is close to 1, which decreases away from the surface. Importantly, the simulations clearly establish a steep temperature gradient (1880 K m^−1^) operating at the top of the NCF@PH. Importantly, this is also quantitatively comparable to the gradient estimated from experiments (1500 K m^−1^), validating the model (Figure 7B–D). Importantly, this reinforces the heat‐blocking mechanism operating in NCF@PH, to impede the dissipation of the solar–thermal energy from the evaporating surface to the bulk water interface.

The average value of the evaporation flux rate (*g*_evap_) is found to be 4.64 kg m^−2^ h^−1^. While the model includes the lowered enthalpy of water evaporation from experimental data (1005 J g^−1^), it does not account for the micromenisci effect at the water‐evaporating interface. This explains the minor deviation between the experimentally observed rate (6.5 kg m^−2^ h^−1^) and that estimated through modeling (4.6 kg m^−2^ h^−1^). It can be observed from Figure 7B–D that there is a localized and high thermal gradient at the top, evaporating surface of the NCF@PH. This is due to the low thermal conductivity of the NCF@PH, as established in both experiment and the current model. Therefore, the low thermal conductivity of the NCF@PH is the dominating factor that creates the large thermal resistance to heat transfer from the top to the bottom surface (across the thickness of NCF@PH). The average temperature of the bottom of the porous surface is found to be 32 °C (Figure 7B–D) and agrees with the experimental observation (34 °C). The negligible difference can be attributed to measurement uncertainty in temperature and evaporative mass, besides the micromenisci effects (**Table** **2**).

**Table 2:** A summarized table of values of key parameters of water evaporation obtained from the experiments and theoretical calculations.

Parameters	Experimental	Simulated
Top surface temperature [°C]	47 (input value)	47 (input value)
Bottom surface temperature [°C]	35	32 (output value)
Evaporation rate [kg m^−2^ h^−1^]	5	4.64 (output)

### 2.9 SunSpring: Completing the Water Cycle

We move on to the next important challenge in sustainable water production, i.e., condensing the evaporated water from NCF@PH3. It is important to note that although a multitude of studies on materials, configurations, and geometries are available on water evaporation,^[^ ^50^, ^51^, ^52^, ^59^, ^60^, ^65^ ^]^ the second half of condensing and collecting the vapor is largely unaddressed and insufficiently investigated. Given the robustness of the NCF@PH3 interfacial evaporator, we therefore proceed to address this critical second half of our artificial hydrological cycle.

**Figure 8:**
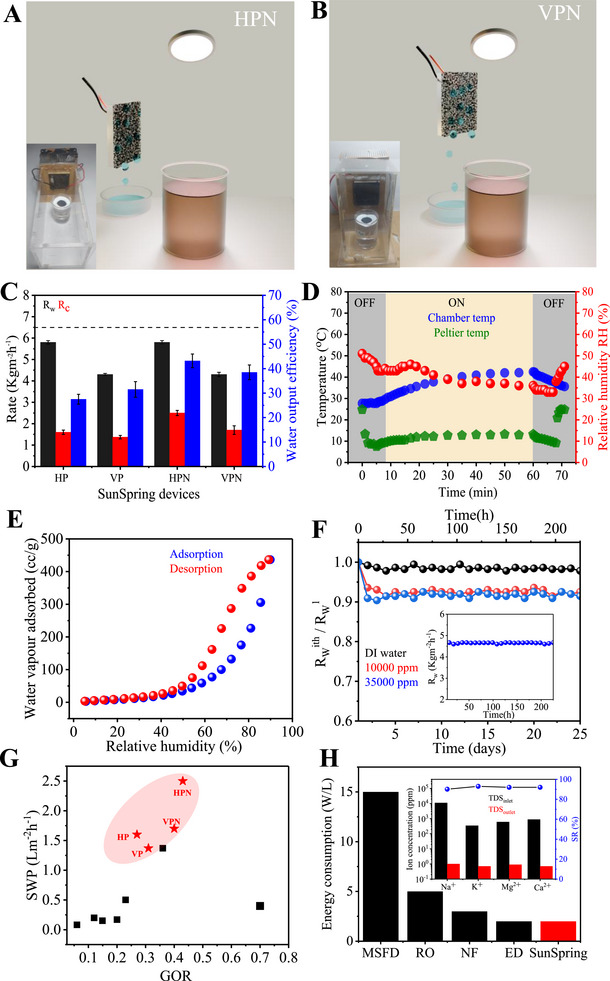
Robust and excellent solar freshwater production by SunSpring. Schematic and photos of A) HPN and B) VPN SunSpring devices, highlighting the relative orientations of the water vapor with respect to the Peltier cooler. C) Comparison of *R*_w_, *R*_c_, and SWP for varying configurations of SunSpring (HP, VP, HPN, and VPN). D) Real‐time monitoring of chamber temperature, Peltier temperature, and relative humidity (RH) in HPN during the experiment, consisting of OFF–ON–OFF solar‐illumination cycle. E) Water vapor adsorption isotherm of NCF at ambient temperature (298 K), indicating low adsorbed water content up to RH 50%. F) Real‐time operation of SunSpring monitored over 225 h, spread over 25 days at Indian Institute of Technology Bombay (19° 07′ 49.76″ N, 72°55′ 03.86″ E) between 5 March 2025 and 29 March 2025. The inset shows the reusability of NCF@PH3 over 25 days (225 h) of usage with 35 000 ppm TDS water feedstock. G) Comparative Ashby plot of SWP and GOR for SunSpring using NCF@PH3, with various other reports in literature.^[^ ^32^, ^34^, ^35^, ^48^, ^49^, ^68^, ^118^ ^]^ H) Overall assessment of energy required for SunSpring, compared to other water purification technologies such as multistage flash distillation (MSFD), reverse osmosis (RO), nanofiltration (NF), and electrodialysis (ED).^[^ ^119^ ^]^ Inset shows the ion concentrations (Na^+^, K^+^, Mg^2+^, and Ca^2+^) of the input simulated seawater and that obtained from SunSpring, after solar–thermal distillation. The corresponding salt‐rejection efficiencies are plotted alongside.

Accordingly, a custom‐built closed transparent setup termed as SunSpring is designed to both evaporate water from a reservoir using NCF@PH3. The chamber is also equipped with a Peltier‐cooled surface (thermo‐electric cooler (TECI)inductively coupled plasma‐atomic emission spectroscopy 12706, 16 cm^2^, and 10 °C at 10 W) to provide the surface for condensing the water vapor (**Figure** **8**A,B). An important aspect of the SunSpring design lies in the decoupling of the sunlight‐admitting surface from the water‐collection surface. In comparison, these two surfaces are the same in conventional solar stills and invariably set up a self‐limiting process, wherein the onset of condensation leads to attenuation of the incident light energy. SunSpring removes this vicious, self‐limiting nature of the process by providing a completely decoupled, colder surface for the condensation of the water vapor. Further, the temperature of the interfacial evaporator and the condensing surface are decoupled and thereby function independent of each other to ensure no kinetic limitations in overall SWP. The water distillate collected from the SunSpring is continuously removed, while appropriately placed humidity and temperature sensors are used to continuously monitor the entire process. Two different geometries of SunSpring are designed and tested for water‐collection efficiency. While the Peltier is placed horizontally at the same height as the interfacial evaporator in the configuration denoted as horizontal Peltier SunSpring (HP), the Peltier is placed at a height of 2 cm above the NCF@PH3 in the vertical‐Peltier SunSpring (VP) configuration (Figure S16a,c, Supporting Information). Thus, while the HP involves lateral movement of the water vapor in a direction perpendicular to the incident light, the VP necessitates vertical transport of water vapor in a direction parallel to the incident light beam. Furthermore, no direct light incidence on the Peltier is possible in both these configurations.

The performance of the system is initially validated by assessing the *R*_w_ in both HP (5.8 kg m^−2^ h^−1^) and VP (4.3 kg m^−2^ h^−1^) configurations (Figure 8C). The *R*_w_ is higher in HP compared to VP and further is similar to that obtained in evaporation‐only studies (6.5 kg m^−2^ h^−1^), owing to the larger dead‐space available (150%) in HP compared to VP. In spite of this difference, the water‐collection rate (*R*_c_) and SWP are similar in both these configurations (≈1.6 L m^−2^ h^−1^, Figure 8C) indicating that the VP is able to more effectively condense the evaporated water. The vertically placed Peltier in VP is naturally aligned with the transport direction of the water vapor and therefore affords a higher *R*_c_, despite a lower *R*_w_. This effectively translates to a slightly improved water‐collection efficiency in VP (GOR = 31.5%), compared to HP (GOR = 27.5%). Overall, the *R*_c_ and SWP from either of these configuration ranges between 1.3 and 1.6 L m^−2^ h^−1^, corresponding to water‐collection efficiency of 27–32%, providing ample scope for improvement (Figure 8C).

The water collection depends not only on the *T*_s_ of the Peltier, but also on the chemistry of the water condensing surface. Herein, a preferred surface should promote water condensation and immediate roll‐off of the water droplets. Accordingly, an optimally hydrophobic and thermally conductive surface coating on the Peltier is expected to enhance the *R*_c_. Given that the NCF coating possesses both these criteria, the SunSpring is modified with NCF‐coated Peltier condensing surface in both the configurations, that are termed as HPN and VPN, respectively (Figure S16b,d, Supporting Information). As expected, the *R*_c_ improved significantly in both HPN and VPN compared to HP and VP, yielding 2.5 and 1.7 L m^−2^ h^−1^ with water collection efficiencies of 43% and 38%, respectively. As expected, such coating does not produce any change in *R*_w_. Furthermore, the enhancement in *R*_c_ is more for HPN at 2.5 L m^−2^ h^−1^, for a water collection efficiency of 43%, when compared to VPN (38%) (Table S9, Supporting Information). Like previously discussed, studies on collection of the evaporated water and translating it to obtain meaningful water distillate are limited. A direct comparison with such reported studies established the *R*_c_ achieved with HPN as being the highest in the class of materials and is also 150% higher than that achieved with commercial solar stills (Figure 8G). The effective decoupling of the light‐admitting surface from the water condensing surface (which are same for conventional solar stills) along with the development of NCF@PH3 as a superior interfacial water evaporator are primary reasons for achieving this performance. Coupled with the high salt‐rejection efficiency of NCF@PH3, the integrated SunSpring shows excellent potential for comprehensive and sustainable solar‐based water purification from underground saline water reserves.

Finally, to understand the higher *R*_c_ and water collection efficiency of HPN over VPN and other systems, we followed the variations in temperature of the condensing surface, chamber, and the RH of the chamber, over the entire process (Figure 8D). The RH of the chamber starts to drop, when the Peltier is switched on, due to the condensing of the ambient water vapor over its surface. At this stage, the solar illumination is not there and, therefore, no additional evaporation of water occurs, leading to saturation of the chamber RH within 30 min. Further, no change in chamber temperature is observed in this stage. With the solar illumination turned on, the Peltier temperature is stable, while the RH and chamber temperatures increase due to the evaporation of the water from the reservoir. Again, the evaporation primarily derives thermal energy from the NCF@PH3, part of which results in radiative and convective heating of the air in the chamber (16%). As a result, a substantial difference of 30–35 °C is produced between the Peltier surface and the chamber temperature, which promotes faster condensation, leading to higher *R*_c_. Furthermore, the NCF surface exhibits minimal water uptake in this range of temperature and RH, as confirmed from the water‐vapor sorption studies (Figure 8E). This reaffirms the hydrophobicity of NCF‐coated Peltier while enabling water condensation over it, and also serves to promote droplet formation and roll‐off. Thus, the capillary condensation process, which is a predominant pathway limiting water collection in porous, high‐surface‐area materials, is minimized with NCF‐coated Peltier, in HPN configuration. The observation of dominant intermediate water on NCF@PH3, as discussed earlier, also substantiates these observations. Finally, in the third stage, when the solar illumination is turned off, the RH and chamber temperature decrease till equilibrium is attained. Such insights present yet another additional aspect of this work by proposing a dynamic condensing surface that can actively adjust to the evolving conditions inside the solar‐water desalination system, to further improve the *R*_c_. To afford a comprehensive analysis, SWP is estimated as per established protocol^[^ ^25^ ^]^

(12) $$\mathrm{SWP}=\frac{E}{L}\alpha \eta_{t}\;\mathrm{GOR}$$

where *E*, *L*, *α*, *η*_t_, and GOR represents solar irradiance (Wm^−2^), latent heat of water evaporation, solar absorptivity of the system, thermal efficiency, and gained‐output ratio, respectively (Section S1.2 in the Supporting Information).

While *R*_c_ represents the analytical water collected from the SunSpring, SWP is a more comprehensive identity that takes all the energetics of a) solar–thermal conversion, b) water evaporation, and c) water condensation into account and thus represent the complete artificial hydrological cycle. Thus, the overall SunSpring operates with one of the highest SWP of 2.5 L m^−2^ h^−1^ with a robust operational stability.

### 2.10 Long‐Term Operability, Stability, Reusability, and Water‐Energy Estimation

To validate the robustness of operation, the setup was subjected to continuous, on‐field experiments lasting for over 25 days and spread over 225 solar hours using highly saline, seawater like (35 000 ppm) water feedstock. The *R*_w_ in the entire range of water salinity (0–35 000 ppm) exhibited no variations across all these 25 days of testing. Moreover, visible salt deposition over the NCF@PH3 was observed at the end of each day, while using seawater like feedstock (35 000 ppm). Since such salt deposition was primarily confined to the external surface of NCF@PH3 as established earlier (vide supra, Figure 4), it provides an important advantage in terms of reusability of the NCF@PH3. Accordingly, the salt deposited on NCF@PH3 could be easily dissolved in water within 5 min, without any agitation. The resulting washed NCF@PH3 membrane, when reused for the next day, exhibits invariant performance, establishing its robustness and providing a clear, facile process for its reusability and long‐term operability (Figure 8F, inset). To further demonstrate the practical viability of SunSpring, experiments using simulated seawater were carried out for extended time periods. The SunSpring was operated for five continuous cycles, with the duration of each cycle being 10 h. After 50 h of such experiments, the salt content of various cations used in the feedstock was reassessed through inductively coupled plasma‐atomic emission spectroscopy (ICP‐AES). This enables us to determine the cation‐dependent salt rejection by NCF@PH3. Importantly, the salt rejection remained consistent over the entire period of testing and showed a uniformly high efficiency of >90% for all the cations tested (Na^+^, K^+^, Mg^2+^, and Ca^2+^; Table S5, Supporting Information). Furthermore, the *R*_w_ remained consistent throughout the duration of the test (50 h), reaffirming the stability of operation (Figure S17, Supporting Information). Finally, the structural and morphological stability of NCF@PH was also retained after such prolonged testing, as observed in SEM–EDS (Figures S20 and S21, Supporting Information).

Given that the maximum amount of energy required for the rate‐determining step of water evaporation is derived from sunlight in HPN‐SunSpring, it becomes essential to compare the overall energy expenditure for the entire artificial and accelerated hydrological cycle occurring inside it. In this context, we acknowledge that while processes such as RO, multistage flash distillation (MSFD), and NF are intrinsically scalable with an energy requirement ranging from 3 to 15 W L^−1^ of pure water output, the proof‐of‐concept demonstration of SS‐HPN achieved here offers clean water at the lowest energy requirement of 2 W L^−1^ (Figure 8H).

The Peltier‐based SS‐HPN can work only during sunlight hours, because it exclusively draws its energy from the sun for the evaporation of water. The role of Peltier is to provide an efficient cold surface that facilitates the condensation of vapor by a) maintaining a constant temperature of the condensing surface and b) minimizing the humidity build up within the chamber. Importantly, this configuration decouples the condensing process from the evaporation process. Consequently, additional experiments carried out by switching OFF the Peltier in SS‐HPN yield significantly low SWP as summarized in Table S9 and Figure S18 (Supporting Information).

The theoretical limit of solar‐based interfacial evaporation rate (*R*_w, max_) is estimated as^[^ ^120^ ^]^

(13) $$R_{w,\max}=Q/\left[L+c_{p}\left(T_{s}-T_{w}\right)\right]$$

given *Q* is the solar flux (W m^−2^), *L* is the latent heat of water evaporation, *C*_p_ is the constant‐pressure specific heat, and *T*_s_ and *T*_w_ are the evaporating surface temperature and bulk water temperatures, respectively. Our experiments clearly confirm the dominance of intermediate water phase at the NCF@PH that substantially lowers the latent heat of evaporation from that of bulk water (2260 J g^−1^) to 1005 J g^−1^. Accounting for this lowered enthalpy, the *R*_w,max_ estimate is 6.26 kg m^−2^ h^−1^. Such an estimate is in excellent agreement with the experimental observations (6.5 kg m^−2^ h^−1^).

This offers interesting scalable opportunities in particularly water scare and economically backward communities that are blessed with abundant sunshine, and can drive a paradigm shift in equitable access of clean water globally.^[^ ^40^ ^]^ In summary, the HPN design of SunSpring combines several aspects of materials, surfaces, and engineering design to achieve a system‐level demonstration of pragmatic water purification and also offers potential interesting alternative for making brine from the surface‐deposited salt with minimal CO_2_ footprint per unit volume of distilled water (Figure S19, Supporting Information).

## 3 Conclusion

In summary, the combination of specifically designed materials and the system demonstrated shows excellent potential for viable solar‐water production with a positive water‐energy nexus. The materials employed (NCF, PH), processes utilized such as spray‐coating, and demonstrations done all point to interesting capabilities for practical scaling. The decoupling of the evaporation step from the condensation step also enables greater flexibility at system levels to achieve higher SWP. SunSpring, consisting of NCF@PH as interfacial evaporator along with a decoupled Peltier‐based condensing surface, has maintained consistent and robust performance with both highly saline water for over 25 days (225 h) and simulated seawater for 5 days (five cycles of 10 h each). The *R*_w_ and SWP estimated from both these extended and aggressive test‐conditions were found to remain consistent over the entire testing phase (4.8 kg m^−2^ h^−1^ and 18 L m^−2^ day^−1^, respectively). We acknowledge that challenges related to biofouling and clogging by particulate matter would persist in real‐world operations. Accordingly, we propose that a prefiltration system can be integrated with SunSpring to minimize such stability‐related issues. Encouraged by these factors, scale‐up demonstrations at the level of 100 L day^−1^ are currently being tested for robustness of performance and ability to handle TDS of up to 10 000 ppm (that is typical for groundwater in arid regions of India). Such opportunities, although challenging, are particularly interesting in water‐scarce and economically backward communities that are blessed with abundant sunshine, and can drive a paradigm shift in equitable access to clean water globally.

## 4 Experimental Section

### Materials

All chemicals and materials (TMPTA, TMPTMP, 1,2‐dichloroethane, and diphenyl (2,4,6‐trimethylbenzoyl)phosphine oxide/2‐hydroxy‐2‐methylpropiophenone) for synthesis of polyHIPE were purchased from Merck. Hypermer B246 was obtained from Croda International. Urea and tetraethyl orthosilicate (TEOS) were acquired from Sigma–Aldrich. Cyclohexane (C_6_H_12_), pentanol (C_5_H_11_OH), cetyltrimethylammonium bromide (CTAB), NaOH, NaCl, and isopropyl alcohol (IPA) were procured from Merck. All chemicals were used without any further purification. All experiments were conducted using Millipore water, unless otherwise specified.

### Synthesis of PH

TMPTA (2.97 g, 0.01 mol), TMPTMP (3.99 g, 0.01 mol), and Hypermer B246 surfactant (0.77 g) were dissolved in 1,2‐dichloroethane (DCE, 7 mL in total) and mixed for 5 min at 350 rpm in a 250 mL two‐neck round‐bottom flask with a D‐shaped polytetrafluroethylene (PTFE), overhead paddle stirrer. The flask was protected from light with aluminum foil. Subsequently, 0.7 mL of the photoinitiator (diphenyl(2,4,6‐trimethylbenzoyl)phosphine oxide/2‐hydroxy‐2‐methylpropiophenone blend) was added to the flask and mixed. 59 mL of MilliQ water was then added using a syringe pump at a flow rate of 45 mL h^−1^. The HIPE solution was then poured into a cylindrical mould between two glass plates, and it was irradiated three times per side with a UV light hammer (belt speed of 1 m min^−1^). The polyHIPE was removed from the mould and washed in a water bath for 24 h. The polyHIPE was then Soxhlet‐extracted with acetone for 24 h, air‐dried, and vacuum‐dried for 24 h to retain the 3D interconnected pore structure generated during the emulsion polymerization and simultaneously remove the water. The dried monolith was sliced with a vibrating microtome to create discs of 200 µm thickness. The standard diameter of such polyHIPE disks was 2 cm and the density was 0.1 g cm^−3^ as measured with a gas pycnometer.

### Synthesis of NCF

Dendritic fibrous nanosilica, synthesized as per established protocol, was employed as a sacrificial template to produce NCF.^[^ ^121^ ^]^ Briefly, acetylene was employed as a gas‐phase carbon source for conformal and uniform deposition of carbon over DNFS, in a thermal chemical vapor deposition system. The deposition was carried out at a flow rate of 100 sccm of acetylene for 10 min. The black powder obtained was treated with 1 m NaOH solution to dissolve the DFNS template and generate an exactly complementary NCF structure.

### Preparation of NCF@PH Photothermal Interfacial Membrane

A uniform dispersion of NCF (5 mg in 2 mL of IPA) was spray‐coated onto the surface of PH, which was preheated to 60 °C. The spray coater was maintained at a fixed distance of 10 cm above the membrane, and spraying durations varied from 0.5 to 3 min. The time of spray coating was varied to result in NCF@PH membranes, termed as NCF@PH1, NCF@PH2, and NCF@PH3 whose surface coverage were 0.12, 0.22, 0.37, 0.52, and 0.6 mg cm^−2^. Further details regarding the methods can be found in the Supporting Information.

## Conflict of Interest

The authors declare no conflict of interest.

## Author Contributions

M.A.V. and A.S. contributed equally to this work. M.A.V. and A.S. conceptualized, performed the materials synthesis and characterizations, data acquisition and evaluation, and co‐wrote the manuscript. M.M. and S.K.S. performed the numerical simulations, and C.N.R. prepared the polyHIPE sheets. C.S. and N.R.C. organized and supervised the whole research, wrote, edited, and validated the manuscript. All authors contributed to the discussion and preparation of the manuscript. C.S., M.A.V., and A.S. discussed the experimental data and co‐wrote the manuscript.

## Supporting information



Supporting Information

## Data Availability

The data that support the findings of this study are available from the corresponding author upon reasonable request.
