# Supplementary material for: Ultrathin, Unsinkable, Janus‐Faced Solar–Thermal Interfacial Evaporator for High‐Throughput Seawater Distillation and Solar‐Water Production
Source: Adv Sci (Weinh). 2025 Oct 21;13(6):e11600. doi: 10.1002/advs.202511600 (PMC12866775; doi:10.1002/advs.202511600)
Supplement: Supplementary file 1 — Supporting Information [file ADVS-13-e11600-s001.docx]

**SUPPORTING INFORMATION**

**Ultra-thin, unsinkable, Janus-faced solar-thermal interfacial evaporator for high-throughput seawater distillation and solar-water production**

*Mohammed Aslam Villan, Amrutha Suresh,* *Mukund Misra, Cintia Naranjo Ruiz, Neil R. Cameron^*^, Sandip Kumar Saha^*^ and Chandramouli Subramaniam^*^*


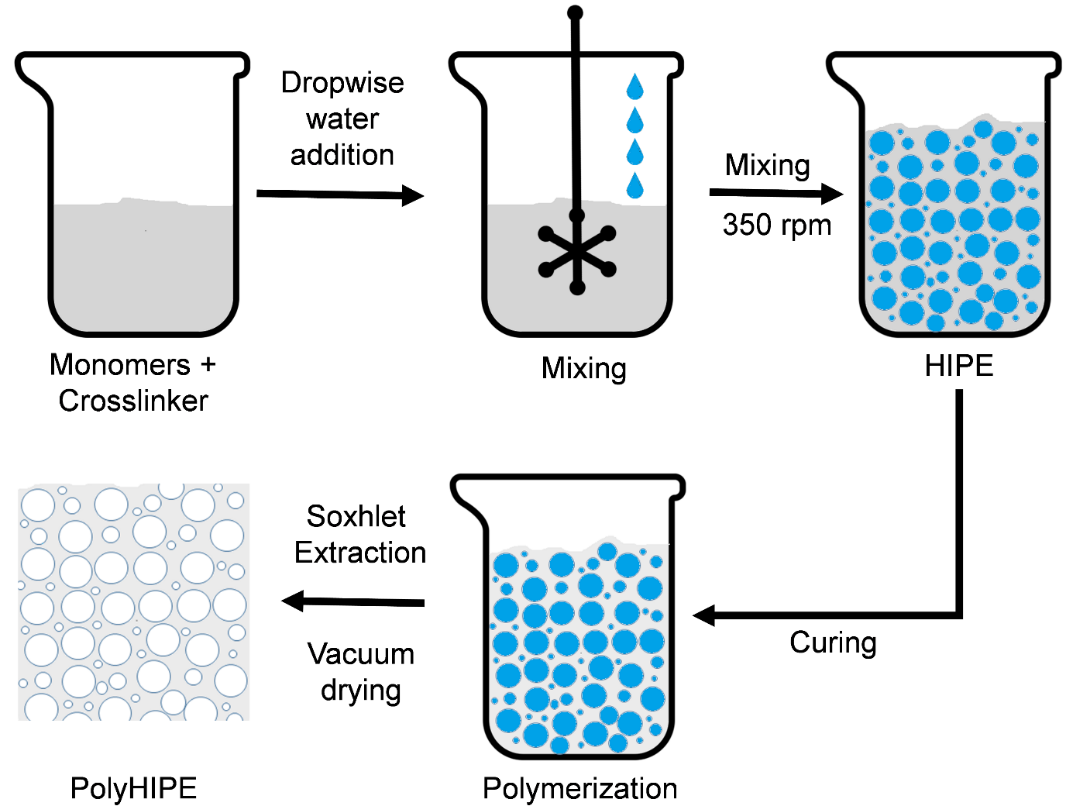


**Figure S1.** Schematic illustration of stepwise synthesis of PH (homo-polymerization of trimethylol propane triacrylate (TMPTA) along with controlled hetero-polymerization of trimethylol propane tris(3-mercaptopropionate, TMPTMP).

**Figure S2.** Characterizations of PH (a) XPS survey spectrum of PH b) Deconvoluted C1s XPS region of PH c) Deconvoluted high resolution S 2P_3/2, ½_ spectra of PH d) ATR-IR spectra of PH and NCF@PH (e, d) SEM images of PolyHIPE.

**Figure S3.** (a) Stress-strain curve of PH obtained from tensile analysis. b) Dynamic mechanical analysis of PH. c) Total reflectance and absorptance of PH over the broad-band region (250 − 2500 nm). d) Thermo-gravimetric analysis of PH.

**Figure S4.** (a,b) SEM images of NCF at different magnifications (inset shows the marigold flower depicting analogy to NCF). (c) TEM image of NCF (d) Total reflectance and absorptance of NCF over a broad-band region (250 − 2500 nm), inset shows the multiple scattering of light at the open-ended microcavities of NCF.

**
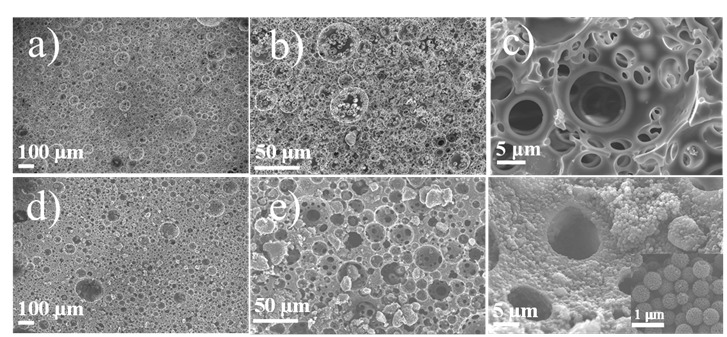
**

**Figure S5.** SEM images of bottom side (PH) (a - c) and top side (NCF@PH) (d - f) at different magnifications of NCF@PH3.

**Figure S6.** (a-d) TEM images of NCF at different magnifications, e) p-XRD profile of NCF, f) Raman spectra of NCF at various excitation wavelengths (532 nm and 633 nm).

**Figure S7.** a) Thermal stress cycles of NCF@PH under solar simulator b) DSC profiles of NCF@PH before and after the heat shock measurements.


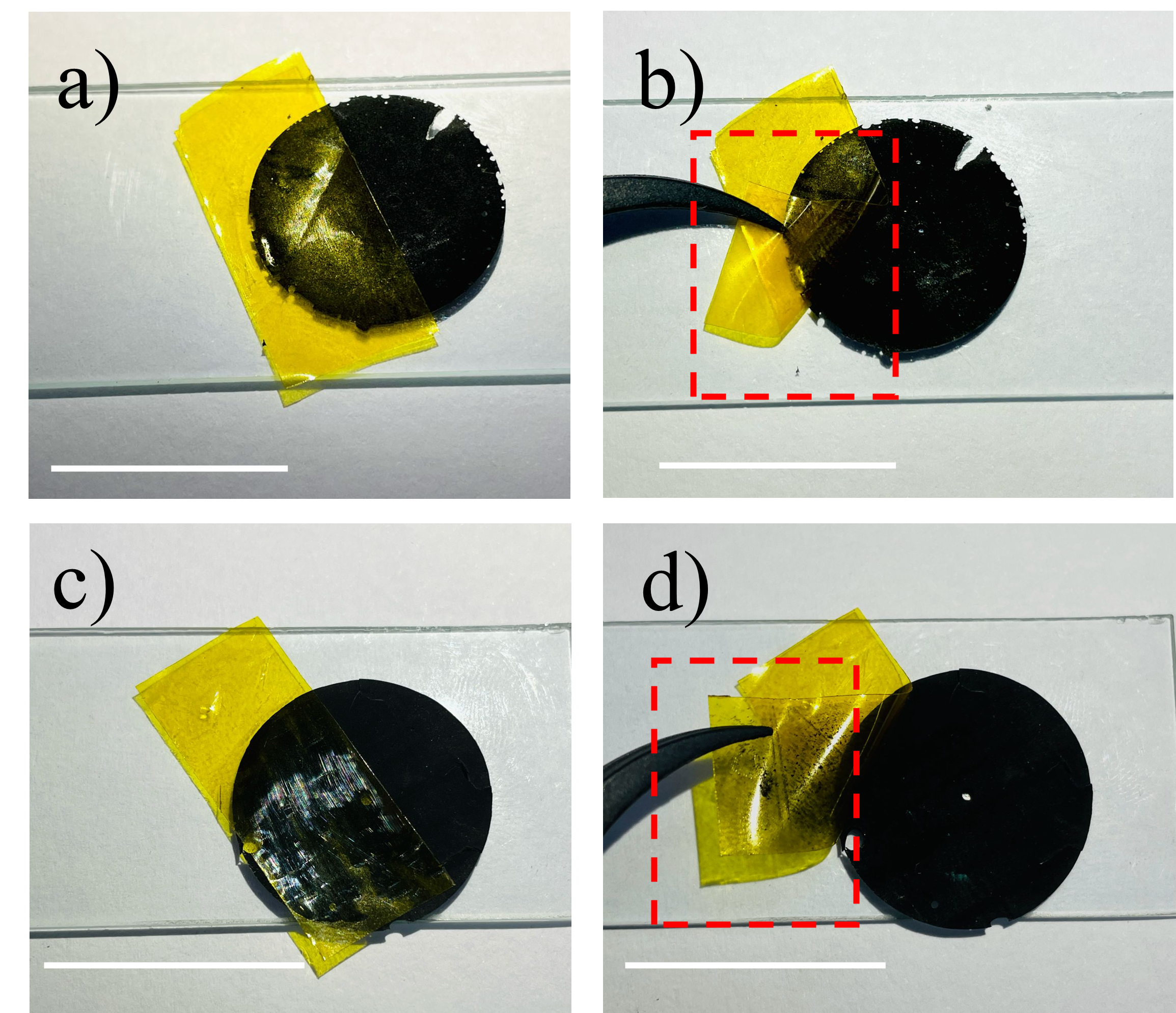


**Figure S8.** Peel-off test of NCF@PH using Kapton tape. (a) Photograph of NCF@PH3 (0.32 mgcm^-2^) before the peel-off test, and (b) after the peel-off test, showing a clean Kapton tape without any residual NCF. (c) Image of NCF@PH4 (0.6 mgcm^-2^) before the peel-off test and (d) after the peel-off test, where NCF traces are observed on the Kapton tape.


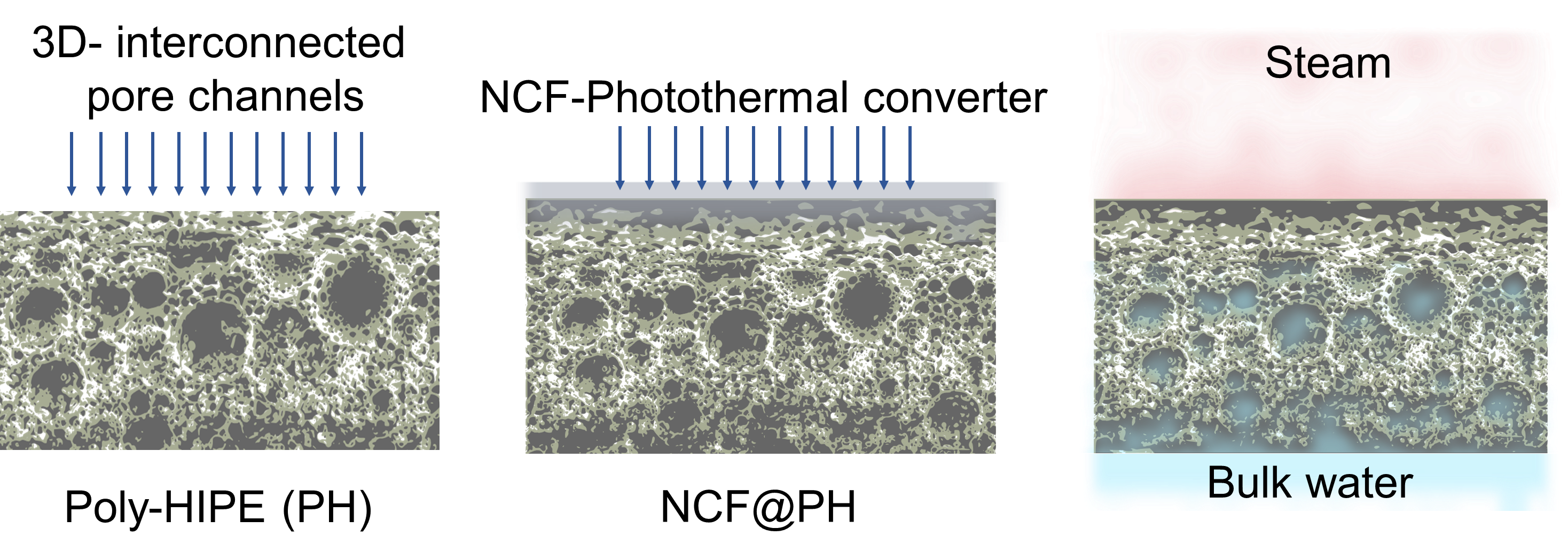


**Figure S9.** Schematic of the cross-section of NCF@PH3 depicting the solar-thermal interfacial evaporation.


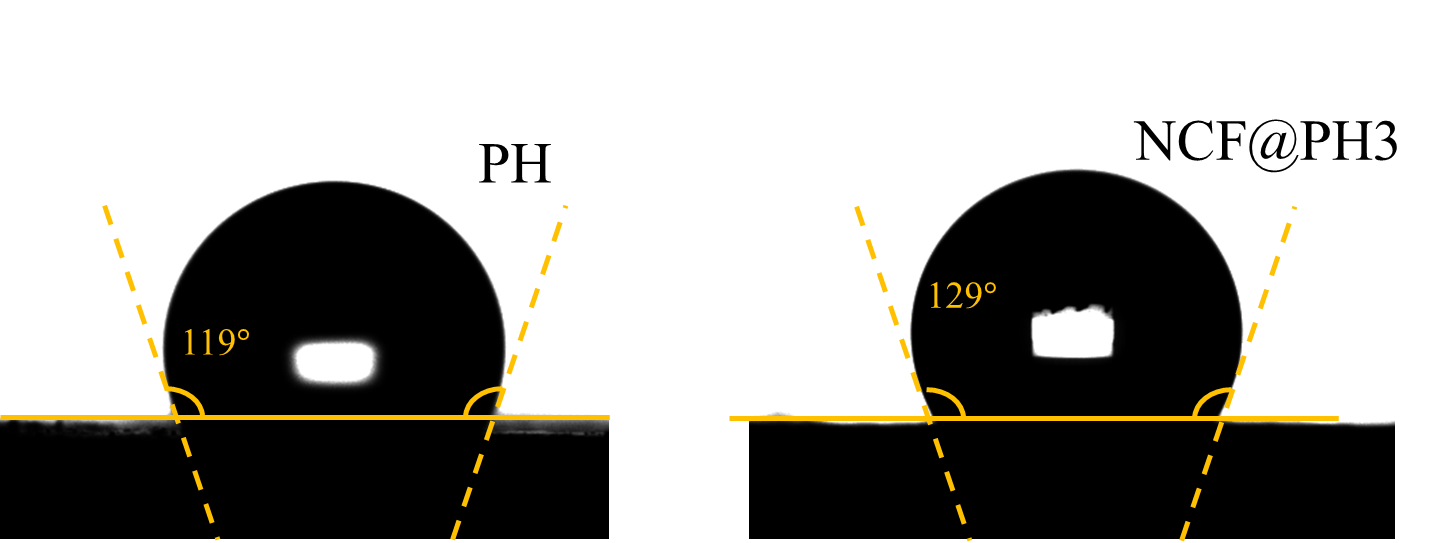


**Figure S10.** Contact angle images PH and NCF@PH with DI water.


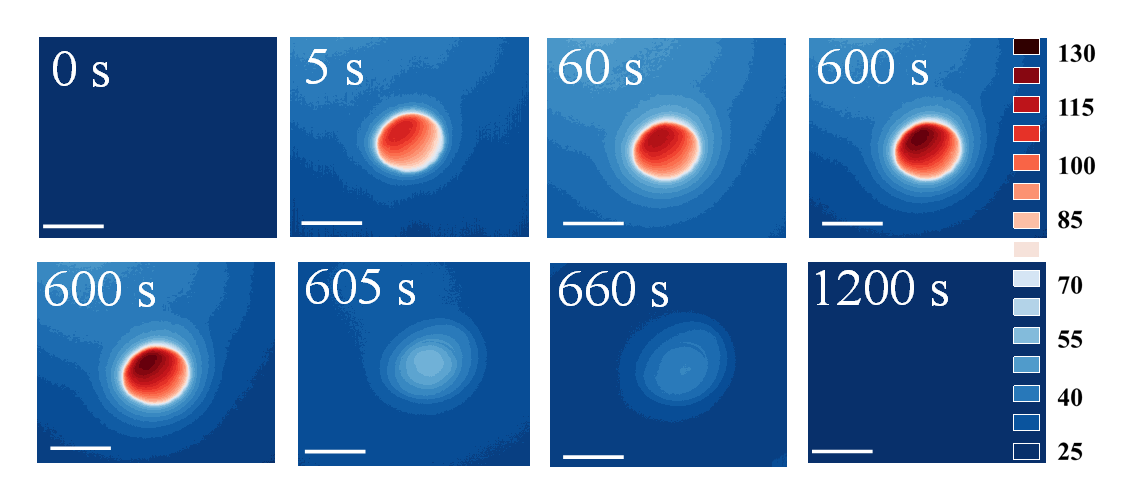
**Figure S11.** Thermal images of NCF@PH3 under solar illumination at different time intervals (0s to 600 s) and light-off from 600s to 1200s.

**Figure S12.** Comparison of thickness of NCF@PH3 with other interfacial evaporators.[1–9]

**Figure S13.** a) Weight change of feedstock with different saline concentrations with time. b) Evaporation rate of feedstock of different saline concentrations.

**Figure S14**. Temperature variation at the surface (*T_s_*) of the NCF@PH evaporator and the bulk temperature (*T_B_*) with time of operation.

**Figure S15.** Raman spectra of PH, PH-H_2_O, and NCF@PH-H_2_O (full spectra).

**Figure S16.** Schematic representation of SunSpring devices (a) HP (b) HPN, (c) VP and (d) VPN.

**Figure S17**. Long-term performance analysis of NCF@PH for 50 h (5 cycles of 10 h each), with simulated seawater. R_w_^ith^ and R_w_^1^ represent the water evaporation rate of the i^th^ hour and the first hour, respectively.


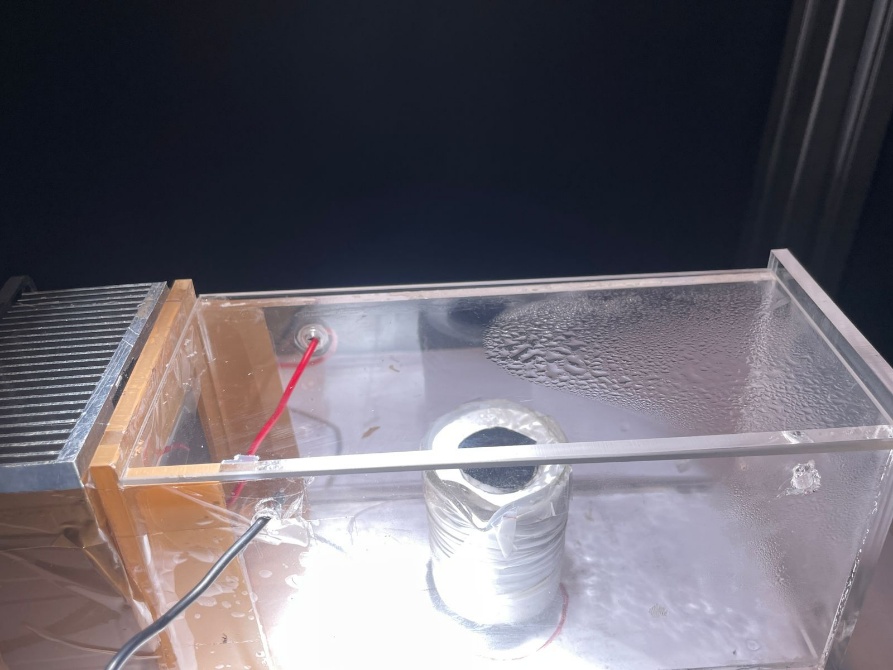


**Figure S18.** Photograph of SunSpring device with Peltier OFF condition. Water vapour condenses over the top surface.

**Figure S19**: Comparison of operational CO_2_ footprint per m^3^ volume of distilled water of SunSpring device (SS) compared to similar state-of-art system used for desalination. RO_SW_ and RO_BW_ refers to reverse osmotic desalination from seawater and brackish water, respectively.


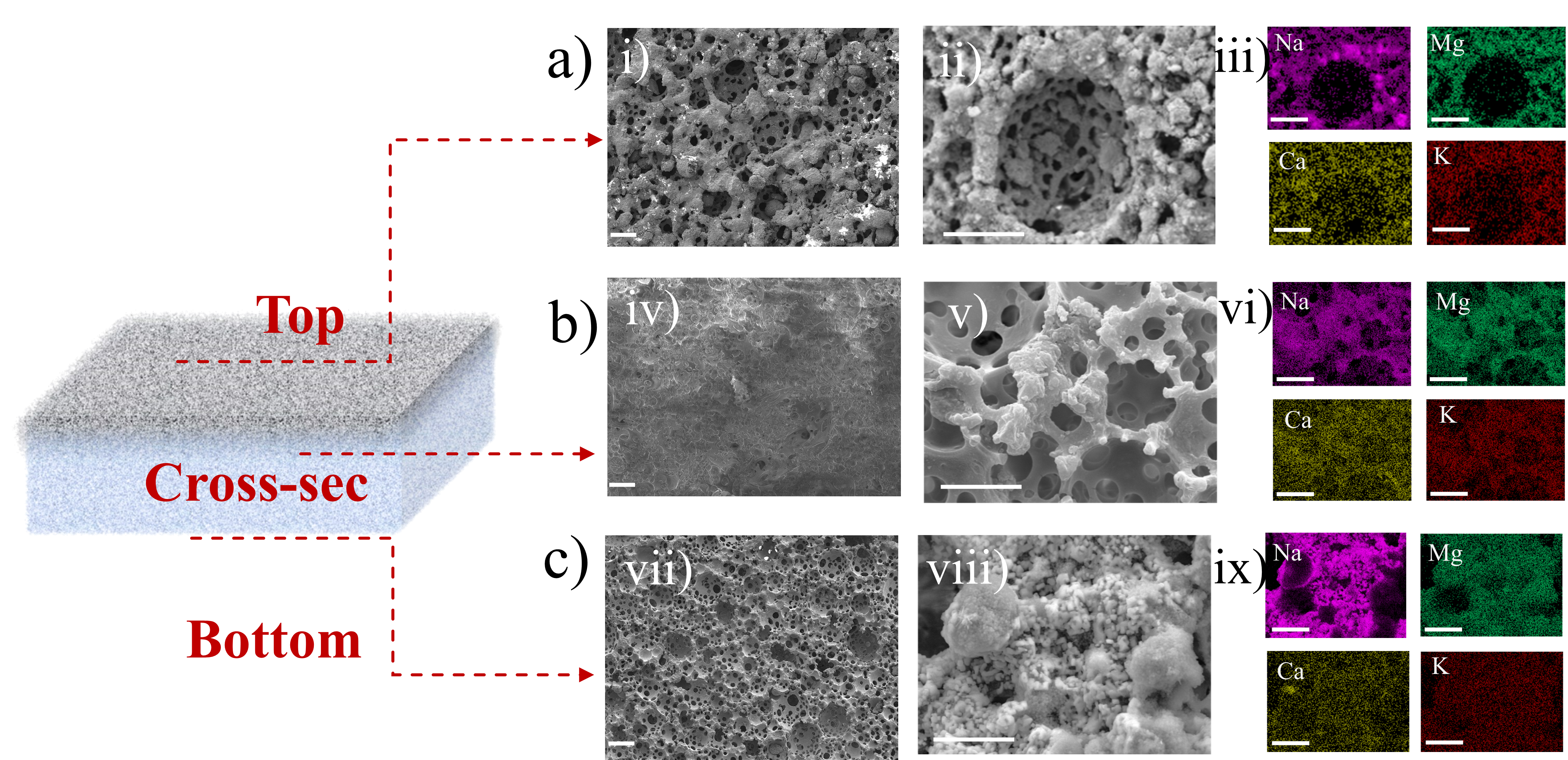


**Figure S20**. SEM images and corresponding EDS elemental analysis (Na, Mg, Ca, and K) of the NCF@PH after 10 h of continuous evaporation. a) SEM images of top side of NCF@PH (i-ii) and corresponding EDS elemental mapping (iii), b) cross-section of NCF@PH (iv-v) and EDS mapping images (vi), and c) bottom side of NCF@PH (vii-viii) and EDS mapping images (ix). All the scale bar is given as 10 µm.

**Figure S21**. SEM images and corresponding Na based EDS elemental mapping of the NCF@PH after 50 h of continuous evaporation. a) SEM images of top side of NCF@PH (i-ii) and corresponding EDS elemental mapping before wash (iii), b) top side of NCF@PH (iv-v) and corresponding EDS elemental mapping after wash (vi), and c) cross-section of NCF@PH (vii-viii) and corresponding EDS elemental mapping before wash (ix). d) cross-section of NCF@PH (x-xi) and corresponding EDS elemental mapping after wash (xii). All the scale bar is given as 10 µm.

**Table S1**. Experimentally observed and theoretically expected parameters used for estimating salt-rejection.

| Initial salinity (ppm) | Time period of evaporation (h) | Expected salinity for 100% theoretical salt-rejection (ppm) | Experimentally observed salinity after 2 h (ppm) |
| --- | --- | --- | --- |
| 5000 | 2 | 5760 | 5658 ± 85.76 |
| 10000 | 2 | 11467 | 11336 ± 41.90 |
| 35000 | 2 | 39417 | 36263 ± 75.76 |

**Table S2.** Total time for saturation and amount of water evaporation from feedstocks of 5000, 10000 and 35000 ppm solutions.

| C_NaCl (aq)_ (× 10^3^ ppm) | Volume of PH (cm^3^) | Volume of NaCl (cm^3^) | Total time for saturation (h) | Amount of expected evaporation (kgm^-2^) |
| --- | --- | --- | --- | --- |
| 5 | 502.72 × 10^-4^ | 83.3 × 10^-6^ | 603 | 3316.5 |
| 10 | 502.72 × 10^-4^ | 88.8 × 10^-6^ | 565 | 2994.5 |
| 35 | 502.72 × 10^-4^ | 1273.14 × 10^-6^ | 39.49 | 181.64 |

**Table S3.** Evaporation rate (R_w_), solar thermal conversion efficiency (η-STC) and solar vapor conversion efficiency (η-SVC) using NCF@PH3 at different salinities.

| C_NaCl (aq)_ (× 10^3^ ppm) | R_w_ (kgm^-2^h^-1^) | η-STC (%) | η-SVC (%) |
| --- | --- | --- | --- |
| 0 | 6.5 | 84.2 ± 0.2 | 99.3 ± 0.5 |
| 5 | 5.5 | 82.2 ± 0.6 | 84 ± 0.4 |
| 10 | 5.3 | 82.5 ± 0.3 | 81 ± 0.5 |
| 35 | 4.67 | 82 ± 0.1 | 71.34 ± 1.2 |

**Table S4.** Summarised TDS values from NCF@PH after 2 h evaporation and corresponding salt rejection efficiency.

| Initial TDS (ppm) | Amount of water evaporated (mL) | TDS obtained from NCF@PH (ppm) | SR (%) = (1-TDS in NCF@PH ÷ Stock TDS) ×100 |
| --- | --- | --- | --- |
| 5000 | 3.6 | 85 | 98 |
| 10000 | 3.5 | 102 | 99 |
| 35000 | 3 | 1400 | 96 |

**Table S5.** Summarized TDS values of stock simulated sea water solution and NCF@PH after 3h, 6h, 10h of evaporation and corresponding salt rejection efficiency of ions.

| Ion | Initial TDS (ppm) | TDS @ 3h  (ppm) | TDS @ 6h  (ppm) | TDS @ 10h  (ppm) | R_w_  (kgm^-2^h^-1^) | SR  (%) |
| --- | --- | --- | --- | --- | --- | --- |
| Na^+^ | 10993 | 12990 | 16480 | 27331 | 4.78 | 92.7 |
| K^+^ | 342 | 392 | 483 | 555 | 4.78 | 93.6 |
| Mg^2+^ | 604 | 729 | 918 | 1404 | 4.78 | 93.4 |
| Ca^2+^ | 879 | 1008 | 1262 | 1880 | 4.78 | 93.8 |

**Table S6.**  Comprehensive literature survey based on various performance matrices (R_w_, R_c_, η-STC, η-SVC, long term stability) of solar-thermal interfacial evaporators and NCF@PH

| **Solar thermal interfacial evaporators** | **R_w_**  **(kg m^-2^h^-1^)** | **R_C_**  **(kgm^-2^h^-1^)** | **η-_STC_**  **(%)** | **η-_SVC_**  **(%)** | **Salt rejection**  **(%)** | **Long-term stability**  **(h)** | **Solar power**  **(kWm^-2^)** | **Ref** |
| --- | --- | --- | --- | --- | --- | --- | --- | --- |
| Carbonized melamine foam | 3 | - | 90 | - | - | 8 | 1 | [10] |
| Kaolin/CNTs/PAM hydrogel | 2.99 | - | - | 73 | - | 9 | 1 | [11] |
| (PVA/PVP)/PDA | 1.6 | - | - | 89 | - | 70 | 1 | [12] |
| Double-interface evaporator-BC_Cu_@FC | 1.57 | - | 87.05 | - | - | - | 1 | [13] |
| CNTs/SiO_2_/PDA@CA/PVB  aerogel | 2.94 | - | - | 91.05 | - | 165 | 1 | [4] |
|  | 5.8 | - | - | 92.83 |  |  | 2 |  |
| Electrospun nanofibrous membrane (PANI@PEN) | 1.82 | - | - | 95.54 |  |  | 1 | [14] |
| Cu dendrites@ CuO | 1.40 | - | - | 87.8 | - | 60 | 1 | [15] |
| PVA-PA/MnO_2_@CC | 3.19 | - | - | 94.1 | - | 135 | 1 | [16] |
| chitosan-based composite aerogel | 1.55 | - | - | 91.47 | - | 18 | 1 | [17] |
| Solid-solid PCMs (BC/PVA/GO-g-LA-SSPCMs) | 3.4 | - | 94.2 | - |  |  | 1 | [18] |
| bilayer-structured solar evaporator (SDWE) | 3.58 | - | - | 93.9 | - | - | 1 | [19] |
| CNF@ZNM-MXene composite | 1.27 | - | - | 82.15 |  |  | 1 | [20] |
| F@CMPsHM-CHM membrane | 1.67 | - | - | - | - | - | 1 | [21] |
| CNT/cellulose hydrogel | 1.52 | - | - | 92 | - | 10 | 1 | [22] |
| Cu based hollow form | 2.284 | - | - | 96.6 | - | 12 | 1 | [23] |
| 3D PPy@D-SCG/PLA | 1.81 | - | 92.7 | - | - | - | 1 | [24] |
| PPy@D-SCG | 1.54 | - | 89.1 | - | - | - | 1 | [24] |
| rGOFpl foam | 1.83 | - | - | 81.4 | - | 12 | 1 | [25] |
| 3D-interconnected CNT hydrogel | 3.55 | - | - | 92 | - | 63 | 1 | [26] |
| rGO-TA-TW | 1.29 | 0.67 | - | - | - | 180 | 1 | [27] |
| Au@Ag-Pd/PS | 3.04 | - | - | 99.1 | - | 40 | 1 | [28] |
| NCF@Filter paper | 5.4 | - | 87 | 186 | - | 270 | 2 | [29] |
| 3D-Fiber aerogel | 2.79 | - | 90.5 | - | - | 24 | 2 | [30] |
| TiO_2_/PPyNP-nanoparticle composite | 2.9 | - | - | 97.3 | - | 100 | 2 | [31] |
| MWCNT/polypropylene/polyethylene | 2.83 | - | - | 91.7 | - | 24 | 2 | [32] |
| CB/PMMA-PAN | 1.3 | - | - | 72 | - | 14 | 1 | [33] |
| CuS/poly(vinylidene fluoride) membrane | 2.91 | - | - | 91.7 | - | 10 | 2 | [34] |
| rGO decorated with magnetic nanoparticles | 2.25 | - | - | 70 | - | - | 2 | [35] |
| MoS_2_-based solar evaporator | 1.95 | - | - | 61 | - | 13 | 2 | [36] |
| Microporous zeolite structures | 1.9 | - | - | 72 | - | - | 2 | [37] |
| **NCF@PH**  **(DI water)** | **6.5** | **2.5** | **84.2** | **99.3** |  |  | **2** | **This work** |
| **NCF@PH**  **(35000 ppm NaCl)** | **4.6** | **1.33** | **84** | **71** | **92** | **> 225** | **2** | **This work** |
| **NCF@PH**  **(Simulated sea water)** | **4.8** | **1.26** | **84** | **75** | **90** | **10** | **2** | **This work** |

**Table S7.** *ΔH_LV_* (J/g) values obtained from DSC

| **Material** | ***ΔH_LV_* (J/g)** |
| --- | --- |
| NCF@PH3 | 0 |
| NCF@PH3-H_2_O | 1005 |
| H_2_O | 2260 |

**Table S8.** Summarized values of the corrected latent heat of vaporization of saline solutions at different TDS and corresponding refined solar-vapor conversion efficiencies (η-SVC).

| Salinity (Wt%) | Latent heat of vaporization  (*∆H_vap_*, J/g) | Rate of evaporation  (Kgm^-2^h^-1^) | Corrected η-SVC  (%) |
| --- | --- | --- | --- |
| 0 | 1005 | 6.5 | 99.7 |
| 0.5 | 999.975 | 5.5 | 84.02 |
| 1.0 | 994.95 | 5.3 | 80.6 |
| 3.5 | 969.8 | 4.67 | 69.38 |

**Table S9.** Performance analysis of different SunSpring devices based on solar water productivity (SWP).

| SunSpring Devices | R_W_ (kg m^-2^ h^-1^) | R_C_ (kg m^-2^ h^-1^) | Water output efficiency (%) | GOR | SWP |
| --- | --- | --- | --- | --- | --- |
| HP | 5.8 | 1.6 | 27.5 | 0.27 | 1.55 |
| VP | 4.3 | 1.37 | 31.5 | 0.31 | 1.79 |
| HPN | 5.8 | 2.5 | 43.2 | 0.43 | 2.48 |
| VPN | 4.3 | 1.7 | 38.5 | 0.38 | 2.19 |
| HPN-Peltier OFF | 5.8 | 0.9 | 15.5 | 0.15 | 0.86 |

**Section 1**

- 1. **Estimation of salt rejection**

Theoretically, a 100% salt-rejection implies zero deposition of salt on the evaporator. It also signifies that all the salt left behind during the water evaporation is returned to the water feedstock, and would result in increase in the salinity of water feedstock. Thus, we estimate the increase in salinity of the water feedstock during our experiments and thereby estimate the salt-rejection from the NCF@PH. Such readings were taken 3 times for each NCF@PH3 sample and averaged over 3 such experiments. Thus, under the conditions of the experiments, 100% salt-rejection implies that the salinity of the water feedstock would be 5760 ppm in 2h, from a starting concentration of 5000 ppm.

Salt rejection (%, SR) was estimated as

$$\mathbf{SR=}\frac{\mathbf{NaCl concentration of feedstock (observed)}}{\mathbf{NaCl concentration of feedstock (theoretical)}}\boldsymbol{\times100}$$

**For 5000 ppm solution,**

After 2h, observed concentration of stock solution = 5658 ppm

Theoretical maximum concentration (if all the salts are rejected by the NCF@PH membrane to stock solution) = 5760 ppm

Salt rejection = 5658 / 5760 = 98.2 %

**For 10000 ppm solution,**

After 2h, observed concentration of stock solution = 11336 ppm

Theoretical maximum concentration = 11467 ppm

Salt rejection = 11336 / 11467 = 98.8 %

**For 35000 ppm solution,**

After 2h, observed concentration of stock solution = 36263 ppm

Theoretical maximum concentration = 39417 ppm

Salt rejection = 36263 / 39417 = 92 %

- 1. **Lifetime assessment of NCF@PH3**

The usable lifetime of NCF@PH3 was estimated based on the salt-rejection and an estimate of the salt-coverage required to fully block the water-transport channels of the NCF@PH3. The amount of salt deposited on NCF@PH3 was estimated by complete dissolution of the salt in DI water, using protocol described earlier. For example,

Weight of salt in PH after evaporation from a 5000 ppm water feedstock = 180×10^-6^ gh^-1^

Volume of NaCl accumulated in NCF@PH3 after 1h = $\frac{mass of the NaCl deposited}{density of NaCl}$

=$\frac{(180 \times{10}^{6}) g}{2.16 g \mathrm{cm}^{-3}}$ =83.3 ×10^-6^ cm^-3^h^-1^

Pore volume of PH disc with 80% porosity (r = 1 cm, h = 200 μm) = 0.8 × πr^2^h = 502.72 × 10^-4^ cm^3^

Total time for saturation of pores by NaCl = $\frac{Volume of NaCl deposited}{Available pore volume of PH}$

= $\frac{502.72 \times{10}^{-4} \mathrm{cm}^{3}}{83.3 \times{10}^{-6} \mathrm{cm}^{3}h^{-1}}$= **603 h**

Weight of salt in PH after 1h (10000 ppm) = 192 µg

Volume of NaCl generated in PH after 1h = $\frac{192 \times{10}^{6} g}{2.16 g \mathrm{cm}^{-3}}$= 88.8 × 10^-6^ cm^3^

Total time for saturation of pores by NaCl = $\frac{502.72 \times{10}^{-4} \mathrm{cm}^{3}}{88.8 \times{10}^{-6} \mathrm{cm}^{3}}$ = **565 h**

Weight of salt in PH after 1h (35000 ppm) = 2750 µg

Volume of NaCl generated in PH after 1h = $\frac{2750 \times{10}^{-6} g}{2.16 g \mathrm{cm}^{-3}}$= 1273.14 × 10^-6^ cm^3^

Total time for saturation of pores by NaCl = $\frac{502.72 \times{10}^{-4} \mathrm{cm}^{3}}{1273.14\times{10}^{-6} \mathrm{cm}^{3}}$ = **39 h**

The volume of freshwater that can be produced during this time is estimated from the SWP.

SWP was estimated as per established protocol^[38]^

$$\mathbf{SWP=}\frac{\mathbf{E}}{\mathbf{L}}\boldsymbol{\alpha\eta t GOR}$$

*E* = solar irradiance

*L* = Latent heat of evaporation

*α* = Solar absorptivity of the system

*η_t_*= Thermal efficiency

GOR = Gained output ratio = R_c_ ÷ R_w_

- 1. **Rate and Efficiency calculations**

**Evaporation and condensation rate calculation**

$$Evaporation rate \left( \mathrm{Kg}m^{-2}h^{-1} \right)=\frac{\text{Weight loss from water feedstock (Kg)}}{\text{Time(h)} \times\text{ Area of illumination (}m^{2}\text{)}}$$

$$Condensation rate \left( Lm^{-2}h^{-1} \right)=\frac{\text{Volume of the distillate collected (L)}}{\text{Time(h)} \times\text{ Area of illumination (m}\text{2}\text{)}}$$

**Solar-thermal conversion efficiency (%)**

$$\boldsymbol{(\eta-STC)=}\frac{\text{P}\text{evaporation}}{\text{P}\text{light}}\boldsymbol{\times100}$$

**𝑃 _𝑙𝑖𝑔ℎ𝑡_ = 𝛼𝐶_𝑜𝑝𝑡_𝑞_𝑖_**

**𝑃 _𝑒𝑣𝑎𝑝_ = 𝑃 _𝑙𝑖𝑔ℎ𝑡_ ― 𝑃_𝑒𝑛𝑣_**

Where, *𝑃_𝑒𝑛𝑣_* is the power density loss during evaporation through radiation and convection

**𝑃_𝑒𝑛𝑣_ = P _radiative_ + P _convective_**

**𝑃_𝑒𝑛𝑣_ = 𝜀𝜎 (𝑇_2_^4^ ― 𝑇_1_^4^) +ℎ (𝑇_2_ ― 𝑇_1_)**

Optical absorption coefficient, *α* = 0.95

Optical concentration, *C_opt_* = 1

Emissivity of the material, *ε* = 0.93

Stefan–Boltzmann constant, *σ* = 5.67 × 10^−8^ W m^-2^ K

Solar flux, *q_i_* = 2000 Wm^-2^

Convection heat transfer coefficient, h = 6.5 W m^-2^ K^-1^

Temperature at the adjacent environment, after illumination *T_1_* = 303 K

Temperature at the evaporating surface after illumination at t = 2h, *T_2_* = 330.27 K

P_radiative_ = 𝜀𝜎 (𝑇_2_^4^― 𝑇_1_^4^) = 158.45 W m ^-2^

Radiative loss (%) = $\frac{P radiative}{P light}\times100$= $\frac{158.45}{2000}=7.9 \%$

P_convective_ =ℎ (𝑇_2_ ― 𝑇_1_) = 156 W m^-2^

Convective loss (%) = $\frac{\mathrm{Pconvective}}{\mathrm{Plight}}\times100$

= $\frac{156.45}{2000}=7.8 \%$

𝑃_𝑒𝑛𝑣_ = 314.45 W m^-2^

𝑃_𝑒𝑣𝑎𝑝_ = 1685.55 W m^-2^

η-STC = $\frac{2000 - 314.45}{2000}=84.2 \%$

**Solar-vapor conversion efficiency (%)**

$$\boldsymbol{(\eta-}\mathbf{SVC}\mathbf{)=}\frac{\text{R}\text{w }\text{(∆H}\text{LV }\text{+ Q)}}{\text{E}\text{light}}\boldsymbol{\times100}$$

R_w_ = Rate of water evaporation

*ΔH_LV_*= Latent heat of evaporation of water

*Q* = Heat delivered to interfacial water = (mc∆T)

*m* = Mass of the evaporated water

*c* = Specific heat capacity of water

*∆T* = Temperature difference

*E_light_* = Solar energy density

$\eta-SVC=\frac{\text{6.5 [ (1}\times\text{106 )+ (0.1 }\times\text{106)]}}{\text{7.2}\times\text{106}}$ ×100 = 99.3%

**Section 2**

- 1. **Instrumentation and characterization**

Field emission scanning electron microscopy (FE-SEM) was performed using Carl-ZEISS Ultra 55, at an accelerating voltage of 3 kV. All the PH samples were coated with Au (5 nm) before the analysis to omit the possibility of charging. Transmission electron microscope (TEM) images were captured with Thermo Scientific, Themis 300 G3, equipped with a BioContinuum GATAN K3 detector. A dilute dispersion of samples in isopropyl alcohol was drop-cast on to Cu TEM grids and mounted after air drying. Three-dimensional optical microscopy images of the sample captured using Keyence VHX-X1 series 3D digital microscope. Three-dimensional X-ray tomography analysis of the samples were acquired using a submicron resolution 3D X-ray microscope (Zeiss, Xradia Versa 520, GeniX3D Cu tube). The images acquired from the analysis were processed and stacked in to a 3D volume viewer using ImageJ software. Thermal images captured using a calibrated FLIR (A6703c) infra-red thermal camera. The camera-sample distance was maintained constant (30 cm) for all the measurements.

Absorptance and reflectance spectra were recorded using Perkin Elmer Lambda 950-UV-Vis-NIR spectrometer equipped with 150 mm integrating sphere. All the textural features of samples were probed using N_2_ and water vapour adsorption isotherms at temperatures of 77K and 298 K respectively, using Autosorb-iQ low-pressure gas-sorption system. All the samples were degassed at 120 ^o^C for 12 h before analysis. Specific surface area calculated using BET equation as

Physisorption of the first adsorbate layer is as follows:

$\frac{x}{W(1-x)}$ = $\frac{1}{C \times W_{ml}}+ \frac{C-1}{C \times W_{ml}} x$ , C = exp ( $\frac{E1-E_{L}}{RT}$)

W = mass adsorbed at relative vapour pressure

x = P/P_0_; P and P_0_ are the actual and saturated vapour pressures of adsorbate

W_ml_ = Mass of adsorbate

Differential Scanning Calorimetry (DSC) analysis of samples were carried out using Rigaku DSC Vesta in nitrogen environment and at a heating rate of 4 ℃min^-1^. Evaporation enthalpy (*∆HL_v_*) of interfacial water and specific heat capacity of the PH are acquired using saphire as standard. Thermal gravimetry analysis (TGA) of samples were recorded using Rigaku Thermogravimetric analyser STA8122 at a heating rate of 5 ℃min^-1^. Samples were pre-heated to 120 ^o^C and subsequently cooled to RT before the analysis for the complete removal of surface adsorbed water and gases. Powder X-ray diffraction trace was obtained using Rigaku, Smartlab model X-ray diffractometer (Cu-Kα radiation, 1.54 Å, step size 0.01) at a scan rate of 5 ^o^ min^-1^. Raman spectra were recorded in Renishaw Laser Raman Imaging System (LRIS) (Invia Reflex, equipped with Peltier cooled CCD detector), with an excitation wavelength of 532 nm, integration time of 5 s at 3 mW laser power. The back-scattered signal was filtered with a Notch filter and dispersed over a grating of 2400 grooves/mm. Contact angle measurements were carried out using GBX Digidrop goniometer after a water drop of 2 μL placed on the sample. The contact angle was measured and averaged over multiple contact-retraction cycles. All the salinity concentrations measured using TOSHCON-TOSHNIWAL -TCM 15 conductivity meter equipped with platinum electrode and thermocouple. Solar-thermal experiments were carried out using Holmarc solar simulator (Xe arc lamp) and the solar intensity measured using photovoltaic power meter. Infrared spectra were recorded in Alpha Bruker ATR-FTIR specrometer, X-ray Photoelectron Spectroscopy carried out for the samples using Kratos Analytical (Axis Supra+) X-ray Photoelectron spectrometer.

**Section 3**

- 1. **Solar-thermal interfacial evaporation experiments**

The NCF@PH3 sample (2 cm diameter, 200 μm thickness) was placed at the air-water interface. Any attempt to push the NCF@PH3 inside the bulk water feedstock, resulted in its spontaneous localization at the interface, due to buoyancy. The rest of the interface was covered with a thick layer of water-impermeable PTFE sheet to avoid any interference (Figure 2B). Further, negligible water evaporation (< 0.3 kgm^-2^h^-1^) was observed from the PTFE covered setup upon solar illumination. The interfacial evaporation setup was then exposed to a controlled solar irradiation of 0.2 Wcm^-2^ and the surface temperature (*T_s_*), bulk water temperature and ambient temperature recorded with an IR thermal camera. The bulk and ambient temperatures were independently verified with thermocouples also and found to be consistent. All the evaporation experiments were conducted for varying time intervals ranging from 20 to 120 minutes, with a consistent interval of 20 minutes between each measurement at a steady-state rate. The weight loss of the water measured using an electronic balance after each experiment.

- 1. **Dynamic water evaporation**

Dynamic water evaporation experiments were conducted using a horizontal shaker with a sinusoidal side-way movement at a frequency of 0.5 Hz. This setup facilitated to simulate real-world conditions of water turbulence. To gain insights into the thermal distribution across the evaporating surface, the surrounding environment, and the bulk water, thermometric videos of the interfacial evaporating system were captured from different angles.

- 1. **TDS Measurements**

The salinity of the feedstock was measured using a conductivity meter after the evaporation process was completed. To estimate the amount of salt deposition on the NCF@PH3, the membrane was carefully transferred into a glass vial containing a known volume (10 mL) of DI water and gently agitated to ensure complete removal of salt from the membrane. Subsequently, the TDS of the resulting solution was measured and used to estimate the amount of salt deposited on the membrane during the evaporation studies.

- 1. **Condensation part of the complete water cycle**

The condensation process in the complete water cycle was carried out using different configurations of SunSpring devices (HP, VP, HPN, and VPN). The light admitting surface of SunSpring was a quartz window. All other sides are made of acrylic. The Horizontal and Vertical in HPN and VPN SunSprings refers to the placement of the Peltier cooled water-condensing surface with respect to the interfacing evaporating surface, as shown in Figure 8 a,b. The Peltier-cooled surface (TECI 12706), operated at 10 W to maintain a constant surface temperature of 10 ± 2 ℃, throughout the studies. The Peltier surfaces in HPN and VPN contained NCF coating on the condensing (cold) side, at a surface coverage of 0.5 mgcm^-2^. The hot-end of the Peltier is attached to a fan-cooled Al heat sink that ensures efficient thermal management. The water condensing Peltier surface was maintained at constant area (4 cm × 4 cm) for all the experiments. The dead-space volume was estimated as the total volume within the device-setup after subtracting the volume occupied by the essential parts, namely, water feedstock, condensate collecting dish and Peltier. Thus the HP and HPN Sunsprings have a dead-space volume of 710 cm³, while it was lower at 407 cm³ for VP and VPN Sunsprings. The surface temperature of the Peltier module and the chamber temperature were continuously monitored using K-type thermocouples, while the relative humidity (RH) inside the chamber was recorded using an RH sensor throughout the evaporation-condensation process.

- 1. **Micro-Raman spectroscopy analysis of water states**

PH and NCF@PH3 were allowed to wick the water from the water surface and immediately transferred to a glass slide placed on the stage of the spectrometer. Raman spectra of the interfacial water of PH and NCF@PH3 acquired using a 532 nm excitation wavelength laser within the spectral range of 3000 cm^-1^ to 3800 cm^-1^.

- 1. **Differential scanning calorimetry studies**

NCF@PH3 containing water was transferred to an DSC analysis pan, crimped carefully and subjected to DSC temperature scans across the temperature range of -20 ℃ to +120 ℃ at a ramping rate of 4 ℃min^-1^ in N_2_ environment. DSC scans with saphire were used as an internal standard.

- 1. **Real-time testing of interfacial evaporation of NCF@PH3**

Experiments on interfacial evaporation were conducted using NCF@PH3 with deionized (DI) water, 10,000 ppm saline solution, and 35,000 ppm saline solution at the rooftop of the Chemistry Department, IIT Bombay (19° 07' 49.76" N, 72° 55' 03.86" E). Testing was performed for 9 solar hours daily from 08:00 to 18:00 hours, for 25 consecutive days. Wind-arrestors were placed on all the sides to prevent interference from wind-currents. The direct normal irradiance was measured using a FLUKE® irradiance meter (IRR1-SOL) and found to be varying between 600 - 800 Wm^-2^, that was in agreement with that provided by National database (www.nsrdb.nrel.gov) during the experimental period.

**REFERENCES**

[1] S. Ma, C.P. Chiu, Y. Zhu, C.Y. Tang, H. Long, W. Qarony, X. Zhao, X. Zhang, W.H. Lo, Y.H. Tsang, Recycled waste black polyurethane sponges for solar vapor generation and distillation, Applied Energy 206, (2017) 63–69. https://doi.org/10.1016/j.apenergy.2017.08.169

[2] S. Chaule, J. Hwang, S.-J. Ha, J. Kang, J.-C. Yoon, J.-H. Jang, Rational Design of a High Performance and Robust Solar Evaporator via 3D-Printing Technology, Advanced Materials 33, (2021) 2102649. https://doi.org/10.1002/adma.202102649

[3] Z. Zhang, C. Xie, Y. Wang, X. Liu, J. Li, H. Chen, J. Wu, X. Wang, L. Wang, Efficient evaporation of seawater desalination by novel double-interface evaporator: Photothermal conversion, water transfer and salt-resistant, Separation and Purification Technology 354, (2025) 128698. https://doi.org/10.1016/j.seppur.2024.128698

[4] R. Wang, J. Deng, P. Wu, Q. Ma, X. Dong, W. Yu, G. Liu, J. Wang, L. Liu, Amphipathic Janus Nanofibers Aerogel for Efficient Solar Steam Generation, Energy & Environmental Materials 7, (2024) e12667. https://doi.org/10.1002/eem2.12667

[5] H. Yan, L. Li, X. Liu, H. Guo, B. Yang, X. Yang, K. Li, Y. Li, Z. Bai, L. Tong, Y. You, P. Wang, A wall-like solar-driven interfacial evaporator fabricated via out-inner anisotropic structure of fresh loofah, Industrial Crops and Products 225, (2025) 120568. https://doi.org/10.1016/j.indcrop.2025.120568

[6] Y. Xue, X. Han, D. Xu, Z. Zhang, L. Zhu, S. Wang, All-in-one chitosan-based aerogel with a semi-clad structure for solar-driven interfacial evaporation, Desalination 575, (2024) 117333. https://doi.org/10.1016/j.desal.2024.117333

[7] Y. Wang, W. Zhao, Y. Lee, Y. Li, Z. Wang, K.C. Tam, Thermo-adaptive interfacial solar evaporation enhanced by dynamic water gating, Nature Communications 15, (2024) 6157. https://doi.org/10.1038/s41467-024-50279-z

[8] Y. Gao, H. Zhao, M. Liu, Y. Wang, Y. Zhang, L. Zhai, X. Liu, F. Zeng, J. Pan, D. Shang, F. Yang, Construction of photothermal hybrid with surface-polarized carbon nanotube twined oxygen-vacancy engineered cuprous oxide for improved PMS activation and water purification, Molecular Catalysis 578, (2025) 114994. https://doi.org/10.1016/j.mcat.2025.114994

[9] M. Qu, Y. Zhao, J. Ge, Y. Xue, L. Mu, Q. Liu, J. Yan, H. Liu, C.-L. Sun, J. He, Multi-Functional Janus Hollow Solar Evaporator Based on Copper Foam for Non-Contact High-Efficiency Solar Interfacial Distillation, ACS Applied Materials & Interfaces 15, (2023) 36999–37010. https://doi.org/10.1021/acsami.3c06049

[10] H. Tahzibi, S. Azizian, Efficient interfacial solar evaporation using a novel carbonized foam as photo-thermal converter, Separation and Purification Technology 354, (2025) 129477. https://doi.org/10.1016/J.SEPPUR.2024.129477

[11] Z. Li, J. Qiu, X. Xu, R. Wan, M. Yao, H. Wang, Z. Zhou, J. Xu, Solar driven kaolin-based hydrogels for efficient interfacial evaporation and heavy metal ion adsorption from wastewater, Separation and Purification Technology 354, (2025) 129243. https://doi.org/10.1016/J.SEPPUR.2024.129243

[12] S. Chaule, J. Hwang, S.-J. Ha, J. Kang, J.-C. Yoon, J.-H. Jang, Rational Design of a High Performance and Robust Solar Evaporator via 3D-Printing Technology, Advanced Materials 33, (2021) 2102649. https://doi.org/10.1002/adma.202102649

[13] Z. Zhang, C. Xie, Y. Wang, X. Liu, J. Li, H. Chen, J. Wu, X. Wang, L. Wang, Efficient evaporation of seawater desalination by novel double-interface evaporator: Photothermal conversion, water transfer and salt-resistant, Separation and Purification Technology 354, (2025) 128698. https://doi.org/10.1016/J.SEPPUR.2024.128698

[14] X. Liu, L. Li, M. Wang, D. Wang, H. Yan, K. Li, Y. Li, Y. Yang, Y. You, X. Yang, P. Wang, In-situ polymerization of PANI nanocone array on PEN nanofibrous membranes for solar-driven interfacial evaporation, Separation and Purification Technology 344, (2024) 127109. https://doi.org/10.1016/j.seppur.2024.127109

[15] H. Zhou, H. Cao, Y. Que, Y. Chen, J. Fu, H. Wang, Y. Zheng, X. He, Y. Yang, J. Liu, Hierarchical Cu dendrites@ CuO multifunctional nanowire mesh for solar-thermal clean water production, Desalination 572, (2024) 117114. https://doi.org/10.1016/j.desal.2023.117114

[16] L. Zou, H. Zhang, Q. Chen, S. Zheng, N. Chen, X. Wu, X. Li, Achieving highly interfacial evaporation rate and continuous salt resistance simultaneously via multi-dimensional composite biomimetic evaporator, Chemical Engineering Journal 498, (2024) 155762. https://doi.org/10.1016/j.cej.2024.155762

[17] Y. Xue, X. Han, D. Xu, Z. Zhang, L. Zhu, S. Wang, All-in-one chitosan-based aerogel with a semi-clad structure for solar-driven interfacial evaporation, Desalination 575, (2024) 117333. https://doi.org/10.1016/j.desal.2024.117333

[18] W. Dong, Y. Wang, Z. Zhang, H. Tao, T. Zhang, G. Meng, J. Wu, S. Jia, Preparation of solid-solid phase change composites and their solar-interfacial water evaporation performance, Journal of Cleaner Production 445, (2024) 141235. https://doi.org/10.1016/J.JCLEPRO.2024.141235

[19] Y. Wang, W. Zhao, Y. Lee, Y. Li, Z. Wang, K.C. Tam, Thermo-adaptive interfacial solar evaporation enhanced by dynamic water gating, Nature Communications 15, (2024) 6157. https://doi.org/10.1038/s41467-024-50279-z

[20] Y. Li, C. Fu, Z. Wang, L. Huang, L. Chen, G. Liao, Q. Zheng, Y. Ni, Novel cellulose-based films with highly efficient photothermal performance for sustainable solar evaporation and solar-thermal power generation, Journal of Cleaner Production 458, (2024) 142416. https://doi.org/10.1016/J.JCLEPRO.2024.142416

[21] W. Luo, J. Zhang, M. Liu, A. Yi, R. Jiao, Z. Zhu, J. Li, H. Sun, A. Li, Excellent solar-driven interface evaporation by an oil repellence Janus photothermal membrane for oily wastewater treatment, Chemical Engineering Journal 483, (2024) 149211. https://doi.org/10.1016/J.CEJ.2024.149211

[22] X. Wang, Y. Sun, G.Y. Zhao, X.Z. Wang, J.S. Qiu, Preparation of carbon nanotube/cellulose hydrogel composites and their uses in interfacial solar-powered water evaporation, New Carbon Materials 38, (2023) 162–172. https://doi.org/10.1016/S1872-5805(22)60621-8

[23] M. Qu, Y. Zhao, J. Ge, Y. Xue, L. Mu, Q. Liu, J. Yan, H. Liu, C.-L. Sun, J. He, Multi-Functional Janus Hollow Solar Evaporator Based on Copper Foam for Non-Contact High-Efficiency Solar Interfacial Distillation, ACS Applied Materials & Interfaces 15, (2023) 36999–37010. https://doi.org/10.1021/acsami.3c06049

[24] C. Shi, X. Zhang, A. Nilghaz, Z. Wu, T. Wang, B. Zhu, G. Tang, B. Su, J. Tian, Large-scale production of spent coffee ground-based photothermal materials for high-efficiency solar-driven interfacial evaporation, Chemical Engineering Journal 455, (2023) 140361. https://doi.org/10.1016/J.CEJ.2022.140361

[25] L. Wang, J. Lin, Y. Li, Y. Yang, X. Liu, Z. Wang, F. Liu, X. Sun, T. Yang, N. Chen, L. Qu, Interfacial charge transfer weakens hydrogen bonds between water molecules to accelerate solar water evaporation, Journal of Material Chemistry A 11, (2023) 7662–7669. https://doi.org/10.1039/D2TA09891A

[26] L. Zhao, Z. Yang, J. Wang, Y. Zhou, P. Cao, J. Zhang, P. Yuan, Y. Zhang, Q. Li, Boosting solar-powered interfacial water evaporation by architecting 3D interconnected polymetric network in CNT cellular structure, Chemical Engineering Journal 451, (2023) 138676. https://doi.org/10.1016/J.CEJ.2022.138676

[27] P. Wu, X. Wu, H. Yu, J. Zhao, Y. Wang, K. Pi, G. Owens, H. Xu, An interfacial solar evaporation enabled autonomous double-layered vertical floating solar sea farm, Chemical Engineering Journal 473, (2023) 145452. https://doi.org/10.1016/J.CEJ.2023.145452

[28] Z. Chen, J. Wang, H. Zhou, Z. Xie, L. Shao, A. Chen, S.-B. Wang, N. Jiang, Janus Nano-Micro Structure-Enabled Coupling of Photothermal Conversion, Heat Localization and Water Supply for High-Efficiency Solar-Driven Interfacial Evaporation, Advanced Functional Materials 33, (2023) 2303656. https://doi.org/https://doi.org/10.1002/adfm.202303656

[29] A. Sah, S. Sharma, S. Saha, C. Subramaniam, Phonon-Engineered Hard-Carbon Nanoflorets Achieving Rapid and Efficient Solar-Thermal Based Water Evaporation and Space-Heating, ACS Applied Materials & Interfaces 15, (2023) 43810–43821. https://doi.org/10.1021/acsami.3c09078

[30] H. Li, H. Wen, J. Li, J. Huang, D. Wang, B.Z. Tang, Doping AIE Photothermal Molecule into All-Fiber Aerogel with Self-Pumping Water Function for Efficiency Solar Steam Generation, ACS Applied Materials & Interfaces 12, (2020) 26033–26040. https://doi.org/10.1021/acsami.0c06181

[31] J. Xing, J. Tong, Y. Liu, Y. Guo, L. Zhuge, D. Zhang, R. Duan, B. Song, Y. Zhao, B. Dong, A high-efficiency ammonia-responsive solar evaporator, Nanoscale 12, (2020) 9680–9687. https://doi.org/10.1039/D0NR00791A

[32] Y. Zhu, G. Tian, Y. Liu, H. Li, P. Zhang, L. Zhan, R. Gao, C. Huang, Low-Cost, Unsinkable, and Highly Efficient Solar Evaporators Based on Coating MWCNTs on Nonwovens with Unidirectional Water-Transfer, Advanced Science 8, (2021) 2101727.https://doi.org/10.1002/advs.202101727

[33] W. Xu, X. Hu, S. Zhuang, Y. Wang, X. Li, L. Zhou, S. Zhu, J. Zhu, Flexible and Salt Resistant Janus Absorbers by Electrospinning for Stable and Efficient Solar Desalination, Advanced Energy Materials 8, (2018) 1702884. https://doi.org/10.1002/aenm.201702884

[34] F. Tao, Y. Zhang, K. Yin, S. Cao, X. Chang, Y. Lei, D. sheng Wang, R. Fan, L. Dong, Y. Yin, X. Chen, Copper Sulfide-Based Plasmonic Photothermal Membrane for High-Efficiency Solar Vapor Generation, ACS Applied Materials & Interfaces 10, (2018) 35154–35163. https://doi.org/10.1021/acsami.8b11786

[35] X. Wang, G. Ou, N. Wang, H. Wu, Graphene-based Recyclable Photo-Absorbers for High-Efficiency Seawater Desalination, ACS Applied Materials & Interfaces 8, (2016) 9194–9199. https://doi.org/10.1021/acsami.6b02071

[36] R. Chen, X. Wang, Q. Gan, T. Zhang, K. Zhu, M. Ye, A bifunctional MoS2-based solar evaporator for both efficient water evaporation and clean freshwater collection, Journal of Material Chemistry A 7, (2019) 11177–11185. https://doi.org/10.1039/C9TA02002K

[37] J. Wang, Z. Liu, X. Dong, C.-E. Hsiung, Y. Zhu, L. Liu, Y. Han, Microporous cokes formed in zeolite catalysts enable efficient solar evaporation, Journal of Material Chemistry A 5, (2017) 6860–6865. https://doi.org/10.1039/C7TA00882A

[38] Z. Wang, T. Horseman, A.P. Straub, N.Y. Yip, D. Li, M. Elimelech, S. Lin, Pathways and challenges for efficient solar-thermal desalination, Science Advances 5, (2025) eaax0763. https://doi.org/10.1126/sciadv.aax0763
